# Allosteric Optical Control of a Class B G‐Protein‐Coupled Receptor

**DOI:** 10.1002/anie.201600957

**Published:** 2016-04-05

**Authors:** Johannes Broichhagen, Natalie R. Johnston, Yorrick von Ohlen, Helena Meyer‐Berg, Ben J. Jones, Stephen R. Bloom, Guy A. Rutter, Dirk Trauner, David J. Hodson

**Affiliations:** ^1^ LMU MunichDepartment of Chemistry and Center for Integrated Protein Science (CIPSM)Butenandtstrasse 5–1381377MunichGermany; ^2^ École Polytechnique Fédérale de Lausanne (EPFL)Institute of Chemical Sciences and Engineering (ISIC)Laboratory of Protein Engineering (LIP)1015LausanneSwitzerland; ^3^ Imperial College LondonSection of Cell Biology and Functional GenomicsDivision of Diabetes, Endocrinology and MetabolismDepartment of Medicine, Hammersmith HospitalDu Cane RoadLondonW12 0NNUK; ^4^ Imperial College LondonSection of Investigative MedicineDivision of Diabetes, Endocrinology and MetabolismUK; ^5^ Institute of Metabolism and Systems Research (IMSR)University of BirminghamB15 2TTUK; ^6^ Centre for EndocrinologyDiabetes and MetabolismBirmingham Health PartnersBirminghamB15 2THUK

**Keywords:** allosteric regulation, beta cells, GLP-1 receptor, photopharmacology, type 2 diabetes

## Abstract

Allosteric regulation promises to open up new therapeutic avenues by increasing drug specificity at G‐protein‐coupled receptors (GPCRs). However, drug discovery efforts are at present hampered by an inability to precisely control the allosteric site. Herein, we describe the design, synthesis, and testing of PhotoETP, a light‐activated positive allosteric modulator of the glucagon‐like peptide‐1 receptor (GLP‐1R), a class B GPCR involved in the maintenance of glucose homeostasis in humans. PhotoETP potentiates Ca^2+^, cAMP, and insulin responses to glucagon‐like peptide‐1 and its metabolites following illumination of cells with blue light. PhotoETP thus provides a blueprint for the production of small‐molecule class B GPCR allosteric photoswitches, and may represent a useful tool for understanding positive cooperativity at the GLP‐1R.

The incretin hormone glucagon‐like peptide‐1 (GLP‐1) is released from enteroendocrine L‐cells in the intestine,^1^ from where it binds cognate receptors to promote the survival of pancreatic beta cells, insulin release, and weight loss.^2^ For these reasons, incretin mimetics based on GLP‐1 have become widely‐prescribed drugs for the restoration of normal glucose levels in type 2 diabetes (T2D),^3^ a socioeconomically costly syndrome affecting almost 400 million individuals worldwide.^4^

The glucagon‐like peptide 1 receptor (GLP‐1R) is a class B G‐protein‐coupled receptor (GPCR) that is primarily coupled to adenylate cyclase activity and 3′‐5′‐cyclic adenosine monophosphate (cAMP) accumulation,^5^ as well as intracellular Ca^2+^ fluxes.^6^ Recently, an allosteric site has been described for this receptor that allows fine modulation of orthosteric ligand binding.^7^ The ligand‐dependent allosteric activator 4‐(3‐(benzyloxy)phenyl)‐2‐(ethylsulfinyl)‐6‐(trifluoromethyl)pyrimidine (BETP) potentiates Ca^2+^ mobilization in response to GLP‐1(7‐36)NH_2_,^7^, ^8^ the active amidated form of GLP‐1. By contrast, BETP amplifies cAMP generation in response to GLP‐1(9‐36)NH_2_,^7^, ^8^ a metabolite and weak partial GLP‐1R agonist. Such interactions are therapeutically desirable, since drugs that target the GLP‐1R allosteric site may improve receptor specificity, thereby reducing side effects.^7^, ^8^, ^9^ However, their investigation is at present hindered by a lack of specific research tools for the fine control of allosterism and receptor movement. Photopharmacology is well‐suited to this task, since it relies on the properties of light to precisely deliver drug activity in space and time.^10^

Herein, we describe the development and testing of PhotoETP, a light‐activated positive allosteric modulator that allows optical control of GLP‐1R signaling and insulin secretion by using blue light (Figure 1 A).

**Figure 1:**
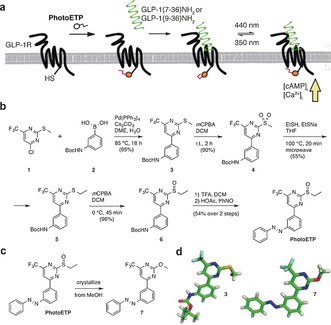
Design and synthesis of PhotoETP. a) An azobenzene unit is installed on the positive allosteric modulator BETP to produce PhotoETP. This allows Ca^2+^ and cAMP responses to GLP‐1 and its inactive metabolites to be potentiated following illumination with UV or blue light. b) Six‐step synthetic pathway for the production of PhotoETP. c) Crystallization of PhotoETP as its methoxy counterpart (**7**) from MeOH. d) Crystal structures for PhotoETP congener **7** (CCDC 1420305 contain the supplementary crystallographic data for this paper. These data can be obtained free of charge from The Cambridge Crystallographic Data Centre) and its precursor bisaryl thioether **3** (CCDC 1420306 contain the supplementary crystallographic data for this paper. These data can be obtained free of charge from The Cambridge Crystallographic Data Centre).

We set out to confer photoswitching on the GLP‐1R allosteric site by subjecting BETP to our “azologization” strategy^11^ (Figure 1 B; see also Figure S1 in the Supporting Information). By coupling commercially available chloropyrimidine **1** and boronic acid **2** under Suzuki–Miyaura conditions, bisaryl thioether **3** was obtained in a yield of 95 %. After oxidizing the sulfur atom with *m*CPBA to its sulfone counterpart **4** in a yield of 90 %, it was exchanged in an aromatic substitution with ethyl sulfide to give ethyl thioether **5** in a yield of 55 %. Subsequent oxidation with one equivalent of *m*CPBA gave access to sulfoxide **6** (96 %), which was deprotected with TFA before undergoing Mills reaction with nitrosobenzene to produce PhotoETP in a yield of 54 % over two steps. Attempts to crystallize PhotoETP from MeOH yielded compound **7**, thus providing further evidence for the electrophilicity of PhotoETP (Figure 1 C). Crystals suitable for X‐ray crystallography were obtained for both **3** and **7** (Figure 1 D).

The UV/Vis spectrum of PhotoETP under illumination at *λ*=440 nm (*trans*, blue) and *λ*=330 nm (*cis*, gray) demonstrated the presence of wavelength‐dependent switching (Figure 2 A), as expected for a *meta*‐azobenzene system (Figure 2 B). Photoswitching could be repeated over several cycles with reasonably fast kinetics (*τ_cis_*=204.2±7.3 s; *τ_trans_*=54.5±2.9 s) and without obvious fatigue (Figure 2 C), and the *cis* and *trans* isomers were separated by LC–MS analysis (Figure 2 D). Together, these features of PhotoETP provide a basis for the allosteric photocontrol of GLP‐1R activity.

**Figure 2:**
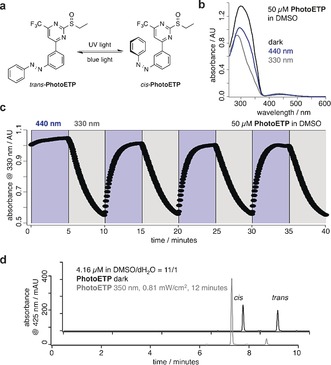
Characterization of PhotoETP. a) Isomerization of PhotoETP between its *trans*‐ and *cis*‐ states with blue light or UV irradiation, respectively. b) UV/Vis spectra of PhotoETP in DMSO following illumination at *λ*=440 nm (blue), *λ*=330 nm (grey), or under dark conditions (black). c) Robust photoswitching between *trans*‐ and *cis*‐PhotoETP induced with *λ*=440 nm and *λ*=330 nm, respectively. d) LC–MS trace of PhotoETP in the dark (black) and after exposure to UV light (*λ*=350 nm; gray).

We next examined whether PhotoETP was able to yield optical control over GLP‐1(9‐36)NH_2_‐induced cAMP generation. Similarly to BETP, *trans*‐PhotoETP potentiated cAMP rises in response to GLP‐1(9‐36)NH_2_ [EC_50_(*trans*‐PhotoETP)=163.3 nm; EC_50_(BETP)=99.5 nm]. Importantly, this activity could be switched off using UV illumination to induce *cis*‐isomer formation (Figure 3 A; unable to calculate EC_50_). The extent of photoswitching is similar to that recently reported for an allosteric modulator of the metabotropic glutamate receptor mGluR5, a class C GPCR.^12^ The effect of PhotoETP on cell viability was determined in islets by using necrosis and apoptosis assays in the dark. At the concentration used throughout the present study, PhotoETP did not induce significant necrosis (Figure 3 B) or apoptosis (Figure 3 C), as measured using propidium iodide incorporation and terminal deoxynucleotidyl transferase dUTP nick end labelling (TUNEL), respectively. By contrast, treatment with staurosporine or thapsigargin positive controls resulted in large increases in necrotic and apoptotic indices (Figure 3 B, C). Furthermore, levels of cleaved caspase‐3, an enzyme involved in the proteolytic cleavage of critical intracellular effectors including poly (ADP‐ribose) polymerase, were unaffected by incubation with PhotoETP (Figure S2).

**Figure 3:**
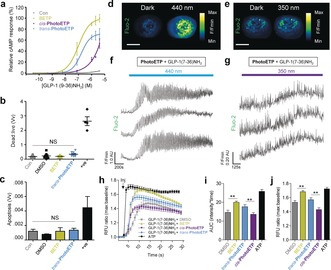
Optical control of cAMP and Ca^2+^ signals. a) Illumination at *λ*=440 nm, but not *λ*=350 nm (UV), optically modulates GLP‐1(9‐36)NH_2_‐induced cAMP generation in CHO‐GLP‐1R cells (*n=*4 repeats). b) Incubation of MIN6 beta cells with PhotoETP for 3 h does not alter cell viability (*n=*8 repeats, +ve=positive control: staurosporine or thapsigargin). c) As for (b) but with apoptosis measured (*n=*3 repeats). d–g) Illumination at *λ*=440 nm, but not *λ*=350 nm, modulates GLP‐1(7‐36)NH_2_‐induced Ca^2+^ increases in intact PhotoETP‐treated islets (*n=*9 recordings from 3 animals). h–j) As for (d)–(g), but in MIN6 beta cells (*n=*8 repeats; RFU=relative fluorescence units, AUC=area under the curve; ATP 10 μM; positive control). In all cases, GLP‐1(7‐36)NH_2_ was co‐applied at 10 nm in the presence of 8–11 mm (islets) or 17 mm (MIN6) d‐glucose. PhotoETP and BETP were applied at 50 μm. NS: non‐significant or **P<0.01 versus *cis*‐PhotoETP or DMSO; one‐way ANOVA. Values represent the mean±SEM. Scale bar, 75 μm.

Next, photoswitching of intracellular Ca^2+^ dynamics was assessed by using PhotoETP directly in beta cells residing within intact islets of Langerhans. Whereas *cis*‐PhotoETP (*λ*=350 nm) showed little effect, the *trans* isomer (*λ*=440 nm) potentiated GLP‐1(7‐36)NH_2_‐induced increases in Ca^2+^ levels (Figure 3 D–G), as previously described for cAMP. The latter could be abolished by using either low glucose (Figure S3A,B) or the specific GLP‐1R antagonist exendin 9–39 (Figure S3C,D). In all cases, results in islets were replicated in MIN6 beta cells subjected to high‐throughput Ca^2+^ screens (Figure 3 H–J). When using batch‐incubated islets, *trans*‐PhotoETP potently amplified GLP‐1(9‐36)NH_2_‐induced insulin secretion (Figure 4 A), whereas *cis*‐PhotoETP was less effective as an allosteric modulator (Figure 4 A). By contrast, BETP, *cis*‐PhotoETP, and *trans*‐PhotoETP all augmented insulin secretion in response to GLP‐1(7‐36)NH_2_ by almost 2‐fold (Figure 4 B), with no evidence of photocontrol.

**Figure 4:**
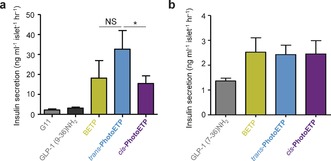
Optical control of insulin secretion. a) *trans*‐PhotoETP is more effective than *cis*‐PhotoETP at potentiating GLP‐1(9‐36)NH_2_‐induced insulin secretion. b) *trans*‐PhotoETP and *cis*‐PhotoETP similarly potentiate GLP‐1(7‐36)NH_2_‐induced insulin secretion. In all cases, PhotoETP and BETP were applied at 50 μm with either GLP‐1(9‐36)NH_2_ 100 nM or GLP‐1(7‐36)NH_2_ 10 nM, as indicated. d‐glucose was present at 11 mM. NS: non‐significant or *P<0.05 versus *cis*‐PhotoETP or BETP; one‐way ANOVA. Values represent the mean±SEM.

PhotoETP was almost 20 % *cis*‐enriched under benchtop conditions (^1^H NMR spectrum; see the Supporting Information), whereas the UV/Vis spectra revealed a more pronounced π–π* band in the dark (see Figure 2 B). This finding can be explained by consulting a model of the glucagon receptor mutant F345C bound to BETP, where a twisted conformation of the benzylether is found to be the lowest energy state.^7^ Such a conformation may also be adopted by *cis*‐PhotoETP owing to its higher affinity for covalent binding. However, in contrast to BETP, which can reorganize its molecular shape in response to orthosteric ligand binding, PhotoETP would remain trapped in its *cis* state until illumination to induce *trans*‐isomerization. Although the exact isomer ratio at the receptor is difficult to determine empirically, such properties may nonetheless afford fine control over photoswitching, with dark conditions, 440 nm illumination, and 350 nm illumination leading to graded Ca^2+^ responses (Figure S4). Further studies using rigid *E*‐ and *Z*‐stilbene bioisosteres of PhotoETP will be required to better delineate the mechanisms involved.

The data presented herein outline a straightforward synthetic strategy for the production of a blue‐light‐activated positive allosteric modulator, which enables photocontrol of GLP‐1R activity through a feedforward loop encompassing the orthosteric site (Figure 1 A). Although similar “alloswitches” have been described for ionotropic and metabotropic mGluRs,^12^, ^13^ this is the first demonstration of their use in a therapeutically relevant class B GPCR. Using a combination of Ca^2+^, cAMP, and insulin assays in CHO‐GLP‐1R and MIN6 cells, as well as islets of Langerhans, we were able to show that PhotoETP allows photoswitching of responses to GLP‐1(7‐36)NH_2_ and its less active breakdown product, GLP‐1(9‐36)NH_2_, with similar potency to native BETP. Notably, PhotoETP displays unusual behavior in cells, where it shows an enriched *cis* content when interacting with its target in the dark. Indeed, the more active *trans* isomer has to be photochemically induced by irradiation with blue light. As a result of these properties, PhotoETP, together with the recently described signal‐biased GLP‐1R photoswitch LirAzo,^14^ may enable the precise dissection of allosteric–orthosteric cooperativity, molecular movement, and binding.

Both BETP and PhotoETP were more effective at potentiating GLP‐1(9‐36)NH_2_‐induced compared to GLP‐1(7‐36)NH_2_‐induced insulin secretion. This suggests that cAMP rather than Ca^2+^ is the primary driver of the “incretin effect”, and is consistent with previous results obtained using LirAzo.^14^ Intriguingly, optical control of insulin release could only be observed in GLP‐1(9‐36)NH_2_‐ and PhotoETP‐treated islets, where blue light provoked a two‐fold higher response than UV illumination. Although the exact reasons for this remain unknown, it may reflect an inability to detect relatively small isomer‐induced differences in intracellular Ca^2+^ versus cAMP concentration at the level of secretion in islets.

BETP was susceptible to UV‐A‐induced but not white‐light‐induced reactions, thus making it a poor control for photoswitching purposes (see Figure S5–S8). In contrast, PhotoETP was remarkably robust. This protective effect stems from the azobenzene unit, which preferentially harvests UV‐A photons with its π–π* band to undergo isomerization. In other words, by installing an azobenzene moiety onto BETP, side reactions can be quenched and the resulting molecule stabilized. Nevertheless, the UV‐A‐induced rearrangement of BETP to its sulfenic ester counterpart via a Meisenheimer complex, and the accompanying transformation, is in itself an interesting finding (Figure S5,S6). Although related rearrangements of sulfoxides have been reported,^15^ sulfenic esters have not been isolated as products owing to the low (UV‐C) wavelengths used in these experiments. Such rearrangements are relevant for drug activity, as best exemplified by acid‐activation of the irreversible proton‐pump inhibitor omeprazole (Prilosec).^16^

In summary, we showcase PhotoETP, a light‐activated modulator for allosteric optical control of GLP‐1R function, and highlight the requirement to run parallel control experiments with benchmark drugs in photopharmacology. PhotoETP, or optimized derivatives thereof, may be useful in drug‐discovery programs aimed at unraveling the complexity of allosterism and class B GPCR signaling.

## Supporting information

As a service to our authors and readers, this journal provides supporting information supplied by the authors. Such materials are peer reviewed and may be re‐organized for online delivery, but are not copy‐edited or typeset. Technical support issues arising from supporting information (other than missing files) should be addressed to the authors.

Supplementary
