# Supplementary material for: Allosteric Optical Control of a Class B G‐Protein‐Coupled Receptor
Source: Angew Chem Int Ed Engl. 2016 Apr 5;55(19):5865–8. doi: 10.1002/anie.201600957 (PMC5031193; doi:10.1002/anie.201600957)
Supplement: Supplementary file 1 — Supplementary [file ANIE-55-5865-s001.pdf]

Angewandte Chemie
Eine Zeitschrift der Gesellschaft Deutscher Chemiker

Supporting Information

Allosteric Optical Control of a Class B G-Protein-Coupled Receptor

Johannes Broichhagen, Natalie R. Johnston, Yorrick von Ohlen, Helena Meyer-Berg, Ben J. Jones, Stephen R. Bloom, Guy A. Rutter, Dirk Trauner, and David J. Hodson**

anie_201600957_sm_miscellaneous_information.pdf

Supporting Information

Table of Contents

1. Synthesis 3

1.1. General 3

1.2. *tert*-Butyl (3-(2-(methylthio)-6-(trifluoromethyl)pyrimidin-4-yl)phenyl)-carbamate (3) 5

1.3. *tert*-Butyl (3-(2-(methylsulfonyl)-6-(trifluoromethyl)pyrimidin-4-yl)phenyl)-carbamate (4) 6

1.4. *tert*-Butyl (3-(2-(ethylthio)-6-(trifluoromethyl)pyrimidin-4-yl)phenyl)-carbamate (5) 7

1.5. *tert*-Butyl (3-(2-(ethylsulfinyl)-6-(trifluoromethyl)pyrimidin-4-yl)phenyl)-carbamate (6) 8

1.6. (*E*)-2-(Ethylsulfinyl)-4-(3-(phenyldiazenyl)phenyl)-6-(trifluoromethyl)-pyrimidine (PhotoBETP) 9

1.7. (*E*)-2-Methoxy-4-(3-(phenyldiazenyl)phenyl)-6-(trifluoromethyl)pyrimidine (7) 11

1.8. 4-(3-(Benzyloxy)phenyl)-2-((ethylthio)oxy)-6-(trifluoromethyl)pyrimidine (8) 12

1.9. 4-(3-(Benzyloxy)phenyl)-2-(ethylsulfinyl)-6-(trifluoromethyl)pyrimidine (BETP) 13

2. Spectra 14

2.1. *tert*-Butyl (3-(2-(methylthio)-6-(trifluoromethyl)pyrimidin-4-yl)phenyl)-carbamate (3) 14

2.2. *tert*-Butyl (3-(2-(methylsulfonyl)-6-(trifluoromethyl)pyrimidin-4-yl)phenyl)-carbamate (4) 15

2.3. *tert*-Butyl (3-(2-(ethylthio)-6-(trifluoromethyl)pyrimidin-4-yl)phenyl)-carbamate (5) 16

2.4. *tert*-Butyl (3-(2-(ethylsulfinyl)-6-(trifluoromethyl)pyrimidin-4-yl)phenyl)-carbamate (6) 17

2.5. (*E*)-2-(Ethylsulfinyl)-4-(3-(phenyldiazenyl)phenyl)-6-(trifluoromethyl)-pyrimidine (PhotoETP) 18

2.6. 4-(3-(Benzyloxy)phenyl)-2-((ethylthio)oxy)-6-(trifluoromethyl)pyrimidine (8) 19

2.7. 4-(3-(Benzyloxy)phenyl)-2-(ethylsulfinyl)-6-(trifluoromethyl)pyrimidine (BETP) 20

3. X-ray Crystallographic Data 21

3.1. *tert*-Butyl (3-(2-(methylthio)-6-(trifluoromethyl)pyrimidin-4-yl)phenyl)-carbamate (3) 21

3.2. (*E*)-2-Methoxy-4-(3-(phenyldiazenyl)phenyl)-6-(trifluoromethyl)pyrimidine (7) 22

4. Experimental methods 23

5. BETP photodegradation 26

6. Supporting Figures 27

7. References 35

1. *Synthesis*

1.1. General

Solvents for chromatography and reactions were purchased in HPLC grade or distilled over an appropriate drying reagent prior to use. If necessary, solvents were degassed either by freeze-pump-thaw or by bubbling N_2 through the vigorously stirred solution for several minutes. Unless otherwise stated, all other reagents were used without further purification from commercial sources.

Flash column chromatography was carried out on silica gel 60 (0.040–0.063 mm) purchased from Merck. Reactions and chromatography fractions were monitored by thin layer chromatography (TLC) on Merck silica gel 60 F254 glass plates. The plates were visualized under UV light at 254 nm or with an appropriate staining method (iodine, *p*-anisaldehyde, $KMnO_4$) followed by heating.

NMR spectra were recorded in deuterated solvents on a BRUKER Avance III HD 400 (equipped with a CryoProbe™) or on a BRUKER Avance III HD 800 instrument and calibrated to residual solvent peaks ($^1H/^{13}C$ in ppm): $CDCl_3$ (7.26/77.00), DMSO-d_6 (2.50/39.52). Multiplicities are abbreviated as follows: s = singlet, d = doublet, t = triplet, q = quartet, br = broad, m = multiplet. Spectra are reported based on appearance, not on theoretical multiplicities derived from structural information.

^{1}H-NMR rearrangement and kinetic studies have been performed in a NMR tube in a DMSO-d_6/D_2O (11/1) mixture illuminated with an UHP-LED-365 (Prizmatic) connected to an AC/DC switching adapter (Mean Well Enterprises). Spectra have been acquired every hour and relevant peaks have been integrated and normalized against the DMSO signal as an internal standard to obtain NMR yields. Fitting of the kinetics was performed by using the linear fit in IgorPro (v6.22, wavemetrics) to obtain the rate constant k_{obs}.

High-resolution and low-resolution electrospray ionization (ESI) mass spectra were obtained on a Varian MAT 711 MS ESI and on an Agilent MS 6120 Single Quad instrument, respectively, operating in either positive or negative ionization modes.

UV/Vis spectra were recorded on a Varian Cary 50 Bio UV-Vis Spectrophotometer using Helma SUPRASIL precision cuvettes (10 mm light path) equipped with a Polychrome V (Till Photonics) monochromator.

LC-MS was performed on an Agilent 1260 Infinity HPLC System, MS-Agilent 1100 Series, Type: 1946D, Model: SL, equipped with a Agilent Zorbax Eclipse Plus C18 (100 x 4.6 mm, particle size 3.5 micron) RP column with a constant flow-rate of 2 mL/min and on a Shimadzu MS2020 connected to a Nexerra UHPLC system equipped with a Waters ACQUITY UPLC BEH C18 (2.1 x 50 mm, particle size 1.7 micron) RP column with a constant flow rate of 0.5 mL/min. Retention times (t_R) are given in minutes (min).

IR spectra were recorded on a *PerkinElmer* Spectrum BX II FT-IR system. The measured wave numbers are reported in cm^{-1}.

Melting points were measured on the apparatus BÜCHI Melting Point B-540 from *BÜCHI Labortechnik AG* or on an EZ-Melt apparatus form *Stanford Research Systems* and are uncorrected.

The data collections were performed on an Bruker AXS TXS diffractometer equipped with a rotating anode generator (**7**, 100 K) and on an Oxford Diffraction Xcalibur diffractometer (**3**, 173 K) using MoKα-radiation (λ = 0.71073 Å) in both experiments.

The structures were solved by direct methods with SIR97[1] and refined by least-squares methods against F^2 with SHELXL-97.[2] All non-hydrogen atoms were refined anisotropically. The carbon-bound hydrogen atoms were placed in ideal geometry riding on their parent atoms while the nitrogen-bound hydrogen in **3** was refined freely. Further details are summarized below.

1.2. *tert*-Butyl (3-(2-(methylthio)-6-(trifluoromethyl)pyrimidin-4-yl)phenyl)-carbamate (3)

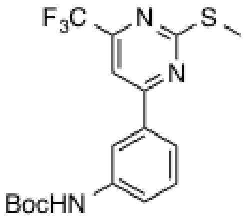

A Schlenk flask was charged under a N_2 atmosphere with 500 mg (2.19 mmol, 1.0 equiv.) of 4-chloro-2-(methylthio)-6-(trifluoromethyl)pyrimidine (**1**), 519 mg (2.19 mmol, 1.0 equiv.) of 3-((*tert*-butoxycarbonyl)amino)phenyl)boronic acid (**2**) and 714 mg (2.19 mmol, 1.0 equiv.) of Cs_2CO_3 and dissolved in a degassed mixture of DME (16 mL) and H_2O (4 mL) before the addition of 254 mg (0.219 mmol, 0.1 equiv.) of $Pd(PPh_3)_4$. The reaction was heated to 85 °C for 18 h before the volatiles were removed *in vacuo* and the crude material was subjected to FCC (pentane/DCM = 1/1 → 0/1) to obtain 805 mg (2.09 mmol) of the desired compound in 95% yield as a white needle-shaped solid.

^{1}H NMR (400 MHz, $CDCl_3$): δ [ppm] = 8.11 (t, *J* = 1.9 Hz, 1H), 7.76 (dt, *J* = 7.7, 1.2 Hz, 1H), 7.61 (m, 2H), 7.43 (t, *J* = 8.0 Hz, 1H), 6.70 (br s, 1H), 2.66 (s, 3H), 1.54 (s, 9H).

^{13}C NMR (101 MHz, $CDCl_3$): δ [ppm] = 174.2, 165.9, 156.3 (q, *J* = 35.8 Hz), 152.6, 139.3, 136.1, 129.8, 128.5, 121.9, 121.9, 120.5 (q, *J* = 275.5 Hz), 117.1, 107.4 (q, *J* = 2.8 Hz), 81.0, 28.3, 14.4.

HRMS (ESI): calc. for $C_{17}H_{19}F_3N_3O_2S^+$ $[M+H]^+$: 386.1145, found: 386.1143.

UV/Vis (LCMS): λ_{max1} = 260 nm; λ_{max2} = 325 nm.

t_R (LCMS; MeCN/H_2O/formic acid = 10/90/0.1 → 90/10/0.1 over 7 min) = 5.585 min.

IR (ATR) in cm^{-1}: 3257, 2982, 2933, 2360, 2340, 1705, 1681, 1591, 1580, 1567, 1539, 1441, 1377, 1366, 1326, 1299, 1282, 1246, 1177, 1144, 1123, 1086, 1056, 999, 978, 959, 911, 896, 873, 863, 853, 794, 774, 704.

m.p.: 124 °C

1.3. *tert*-Butyl (3-(2-(methylsulfonyl)-6-(trifluoromethyl)pyrimidin-4-yl)phenyl)-carbamate (4)

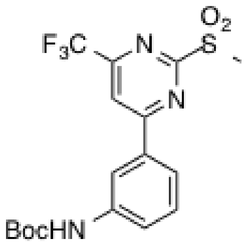

A round bottom flask was charged with 200 mg (0.52 mmol, 1.0 equiv.) of **3** dissolved in DCM (10 mL) before the addition of 349 mg (1.56 mmol, 3.0 equiv.) of *m*CPBA (77%). The reaction was stirred for 2 h at r.t. before the crude material was subjected to FCC (100% DCM) to obtain 195 mg (0.47 mmol) of the desired product in 90% yield as a white oily wax.

^{1}H NMR (400 MHz, $CDCl_3$): δ [ppm] = 8.26 (t, J = 1.9 Hz, 1H), 8.18 (s, 1H), 7.91–7.84 (m, 1H), 7.71–7.67 (m, 1H), 7.48 (t, J = 8.0 Hz, 1H), 6.82 (br s, 1H), 3.48 (s, 3H), 1.54 (s, 9H).

^{13}C NMR (101 MHz, $CDCl_3$): δ [ppm] = 168.8, 166.7, 157.9, 157.3 (q, J = 37.5 Hz), 139.8, 134.4, 134.2, 130.2, 123.3, 122.3, 117.4, 114.8 (q, J = 2.7 Hz), 81.3, 39.1, 28.3.

HRMS (ESI): calc. for $C_{17}H_{17}F_3N_3O_4S^-$ [M-H]$^-$: 416.0897, found: 416.0898.

UV/Vis (LCMS): λ_{max1} = 234 nm; λ_{max2} = 287 nm.

***t*$_R$** (LCMS; MeCN/H_2O/formic acid = 10/90/0.1 → 90/10/0.1 over 7 min) = 4.591 min.

IR (ATR) in cm^{-1}: 3354, 2978, 2930, 2362, 1720, 1587, 1528, 1494, 1443, 1380, 1314, 1277, 1237, 1206, 1143, 1060, 963, 885, 799, 745, 705, 682.

1.4. *tert*-Butyl (3-(2-(ethylthio)-6-(trifluoromethyl)pyrimidin-4-yl)phenyl)-carbamate (5)

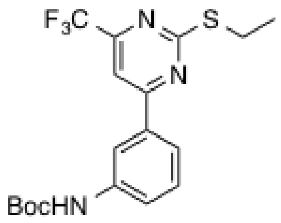

A microwave vial was charged with 580 mg (1.39 mmol, 1.0 equiv.) of **4** dissolved in a mixture of THF (15 mL) and EtSH (2.0 mL) before the addition of 117 mg (1.39 mmol, 1.0 equiv.) of EtSNa. The reaction was heated in a microwave for 30 min at 100 °C before the crude material was subjected to FCC (pentane/DCM = 1/1) to obtain 303 mg (0.76 mmol) of the desired product in 55% yield as a yellowish wax.

^{1}H NMR (400 MHz, $CDCl_3$): δ [ppm] = 8.12 (d, J = 2.0 Hz, 1H), 7.80–7.72 (m, 1H), 7.61 (m, 2H), 7.44 (t, J = 7.9 Hz, 1H), 6.67 (br s, 1H), 3.26 (q, J = 7.3 Hz, 2H), 1.54 (s, 9H), 1.46 (t, J = 7.3 Hz, 3H).

^{13}C NMR (101 MHz, $CDCl_3$): δ [ppm] = 174.0, 165.9, 156.3 (q, J = 35.8 Hz), 152.6, 139.3, 136.2, 129.8, 121.9, 121.8, 119.1, 117.1, 107.4 (q, J = 2.5 Hz), 81.0, 28.3, 25.7, 14.3.

HRMS (ESI): calc. for $C_{18}H_{21}F_3N_3O_2S^+$ $[M+H]^+$: 400.1301, found: 400.1301.

UV/Vis (LCMS): λ_{max1} = 264 nm; λ_{max2} = 326 nm.

t_R (LCMS; MeCN/H_2O/formic acid = 10/90/0.1 → 90/10/0.1 over 7 min) = 5.821 min.

IR (ATR) in cm^{-1}: 3333, 2978, 2931, 1703, 1583, 1544, 1493, 1442, 1378, 1298, 1255, 1154, 1126, 1058, 853, 795, 775, 750, 707.

m.p.: 123 °C

1.5. *tert*-Butyl (3-(2-(ethylsulfinyl)-6-(trifluoromethyl)pyrimidin-4-yl)phenyl)-carbamate (6)

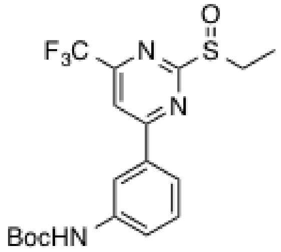

A round bottom flask was charged with 30.5 mg (0.076 mmol, 1.0 equiv.) of **5** dissolved in DCM (4 mL). The reaction mixture was cooled to 0 °C before the addition of 17.1 mg (0.076 mmol, 1.0 equiv.) of *m*CPBA (77%). The reaction was stirred for 45 min while allowing to warm to r.t. before the crude material was subjected to FCC (DCM/MeOH = 100/0 → 100/3) to obtain 30.4 mg (0.073 mmol) of the desired product in 96% yield as a yellow wax.

^{1}H NMR (400 MHz, DMSO-d$_6$): δ [ppm] = 8.25 (s, 1H), 8.04 (s, 1H), 7.90–7.83 (m, 1H), 7.77–7.66 (m, 1H), 7.45 (t, J = 8.0 Hz, 1H), 6.96 (s, 1H), 3.31 (dt, J = 14.7, 7.4 Hz, 2H), 3.19 (dt, J = 13.5, 7.3 Hz, 1H), 1.52 (s, 9H), 1.35 (t, J = 7.4 Hz, 3H).

^{13}C NMR (101 MHz, DMSO-d$_6$): δ [ppm] = 174.4, 167.9, 157.3 (q, J = 36.9 Hz), 152.7, 139.8, 134.7, 130.1, 122.9, 122.2, 120.2 (q, J = 275.8 Hz) 117.4, 112.6 (q, J = 2.7 Hz), 81.0, 47.6, 28.3, 6.4.

HRMS (ESI): calc. for $C_{18}H_{20}F_3N_3NaO_3S^+$ $[M+Na]^+$: 438.1070, found: 438.1073.

UV/Vis (LCMS): λ_{max1} = 237 nm; λ_{max2} = 288 nm; λ_{max3} = 332 nm.

***t*$_R$** (LCMS; MeCN/H_2O/formic acid = 10/90/0.1 → 90/10/0.1 over 7 min) = 4.538 min.

IR (ATR) in cm^{-1}: 3263, 3070, 2979, 2935, 2362, 1720, 1584, 1526, 1494, 1442, 1379, 1300, 1279, 1238, 1149, 1124, 1057, 1026, 996, 985, 882, 842, 798, 749, 704.

m.p.: 66 °C

1.6. (*E*)-2-(Ethylsulfinyl)-4-(3-(phenyldiazenyl)phenyl)-6-(trifluoromethyl)-pyrimidine (PhotoBETP)

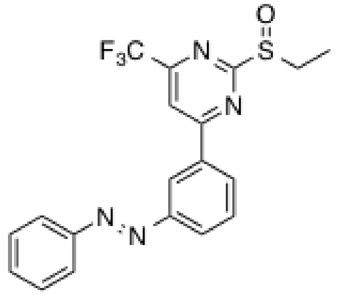

A round bottom flask was charged with 20.0 mg (48.2 µmol, 1.0 equiv.) of **6** dissolved in a mixture of DCM (2 mL) and TFA (2 mL). The reaction was stirred for 1 hour before all volatiles were removed *in vacuo* and the resulting material was dissolved in HOAc (2 mL) and DCM (2 mL) and 15.0 mg (140 µmol, 2.9 equiv.) of PhNO was added in one portion. The mixture was stirred for 18 h at r.t. before it was quenched with an aqueous sat. solution of $NaHCO_3$ and extracted with DCM. The organic layer was dried over $MgSO_4$ and the crude material was subjected to FCC (pentane/DCM = 1/1 → 0/1) to obtain 10.6 mg (26.2 µmol) of the desired product in 54% yield over two steps as a viscous orange oil. NMR data is given for the major *trans*-isomer.

^{1}H NMR (400 MHz, $CDCl_3$): δ [ppm] = 8.68 (s, 1H), 8.44–8.39 (m, 1H), 8.19–8.12 (m, 2H), 8.00–7.94 (m, 2H), 7.72 (t, *J* = 7.8 Hz, 1H), 7.58–7.49 (m, 3H), 7.24 (s, 1H), 3.41–3.29 (m, 1H), 3.29–3.14 (m, 1H), 1.37 (t, *J* = 7.4 Hz, 3H).

^{13}C NMR (101 MHz, $CDCl_3$): δ [ppm] = 175.0, 167.6, 157.6 (q, *J* = 37.0 Hz), 153.2, 152.4, 135.3, 131.7, 130.3, 130.1, 129.2, 126.7, 123.1, 122.5, 120.2 (q, *J* = 275.9 Hz), 112.8 (q, *J* = 2.7 Hz), 47.7, 6.5.

^{19}F NMR (376 MHz, $CDCl_3$): δ [ppm] = –69.4.

NMR revealed a 1/4 ratio of *cis*-/*trans*-AzoBETP.

HRMS (ESI): calc. for $C_{19}H_{16}F_3N_4OS^+$ $[M+H]^+$: 405.0991, found: 405.0993.

UV/Vis (LCMS): λ (*trans*-AzoBETP, $\pi \rightarrow \pi^*$) = 302 nm; λ (*cis*-AzoBETP, n $\rightarrow \pi^*$) = 428 nm.

t_R (LCMS; MeCN/H_2O/formic acid = 10/90/0.1 $\rightarrow$ 90/10/0.1 over 7 min) = 4.149 min (*cis*-AzoBETP), 4.979 min (*trans*-AzoBETP).

IR (ATR) in cm^{-1}: 3065, 2967, 2933, 2874, 1720, 1582, 1528, 1439, 1407, 1379, 1317, 1298, 1260, 1179, 1147, 1124, 1068, 999, 985, 884, 841, 804, 765, 709, 688.

1.7. (*E*)-2-Methoxy-4-(3-(phenyldiazenyl)phenyl)-6-(trifluoromethyl)pyrimidine (7)

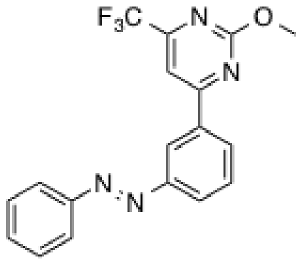

Crystals of (*E*)-2-methoxy-4-(3-(phenyldiazenyl)phenyl)-6-(trifluoromethyl)-pyrimidine **7** were obtained by letting a concentrated solution of **PhotoETP** in MeOH slowly evaporate at r.t. over one week. No additional spectroscopic data was collected.

1.8. 4-(3-(Benzyloxy)phenyl)-2-((ethylthio)oxy)-6-(trifluoromethyl)pyrimidine (8)

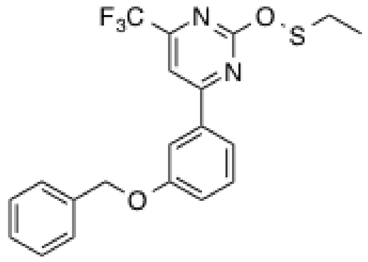

8 was obtained by irradiating a sample of BETP in DMSO-d_6/D_2O (11/1) over 4 h with UV light (365 nm) in 78% yield based on signal integration. After submitting the sample to either FCC (Et_2O/DCM = 9/1) or RP-HPLC (MeCN/H_2O/FA = 10/90/0.1 → 80/20/0.1 over 82 minutes) **8** was obtained as a slight yellow gum that is unstable in solution.

^{1}H NMR (400 MHz, DMSO-d_6): *δ* [ppm] = 8.19 (s, 1H), 7.88–7.86 (m, 2H), 7.53–7.44 (m, 3H), 7.42–7.36 (m, 2H), 7.36–7.32 (m, 1H), 7.29–7.24 (m, 1H), 5.20 (s, 2H), 3.20 (q, *J* = 7.3 Hz, 2H), 1.36 (t, *J* = 7.3 Hz, 3H).

^{13}C NMR (201 MHz, DMSO-d_6): *δ* [ppm] = 172.9, 166.7, 159.2, 137.1, 136.4, 130.9, 128.9, 128.3, 128.1, 120.6, 119.5, 113.8, 69.8, 25.3, 14.5. Missing 3 carbons due to fluorine coupling and resulting smaller quartet peaks diminishing in the baseline (*cf.* with BETP ^{13}C).

^{19}F NMR (376 MHz, DMSO-d_6): *δ* [ppm] = –68.7.

HRMS (ESI): calc. for $C_{20}H_{18}F_3N_2O_2S^+$ [M+H]$^+$: 407.1036, found: 407.1037, calc. for $C_{18}H_{12}F_3N_2O_2^-$ [M–EtS]$^-$: 345.0856, found: 345.0862, calc. for $C_{13}H_{10}F_3N_2OS^-$ [M–OBn]$^-$: 299.0471, found: 299.0475.

UV/Vis (LCMS): λ_{max} = 342 nm.

$\boldsymbol{t_R}$ (LCMS; MeCN/H_2O/formic acid = 10/90/0.1 → 100/00/0.1 over 10 min) = 7.629 min.

IR (ATR) in cm^{-1}: 2929, 2583, 1542, 1493, 1442, 1378, 1296, 1254, 1209, 1179, 1149, 1126, 1026, 854, 786, 737, 708, 696.

1.9. 4-(3-(Benzyloxy)phenyl)-2-(ethylsulfinyl)-6-(trifluoromethyl)pyrimidine (BETP)

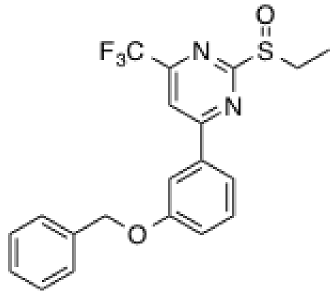

^{1}H NMR (400 MHz, DMSO-d_6/D_2O): δ [ppm] = 8.65 (s, 1H), 8.18–7.88 (m, 2H), 7.56–7.45 (m, 3H), 7.40 (ddd, *J* = 8.0, 7.1, 1.0 Hz, 2H), 7.36–7.29 (m, 2H), 5.22 (s, 2H), 3.30 (dq, *J* = 13.5, 7.4 Hz, 1H), 3.12 (dq, *J* = 13.6, 7.3 Hz, 1H), 1.14 (t, *J* = 7.3 Hz, 3H).

^{13}C NMR (101 MHz, DMSO-d_6/D_2O): δ [ppm] = 173.8, 167.5, 159.3, 137.0, 135.8, 131.0, 128.9, 128.4, 128.3, 121.1, 120.0, 114.4, 70.0, 46.9, 6.2. Missing 3 carbons due to fluorine coupling and resulting smaller quartet peaks diminishing in the baseline.

HRMS (ESI): calc. for $C_{20}H_{18}F_3N_2O_2S^+$ $[M+H]^+$: 407.1036, found: 407.1036.

UV/Vis (DMSO): λ_{max} = 298 nm.

t_R (LCMS; MeCN/H_2O/formic acid = 10/90/0.1 → 100/00/0.1 over 10 min) = 8.272 min.

IR (ATR) in cm^{-1}: 3065, 1580, 1531, 1494, 1444, 1417, 1374, 1325, 1313, 1277, 1262, 1228, 1216, 1198, 1182, 1142, 1124, 1095, 1079, 1070, 1008, 992, 909, 876, 848, 800, 781, 748, 704, 680, 662.

2. *Spectra*

2.1. *tert*-Butyl (3-(2-(methylthio)-6-(trifluoromethyl)pyrimidin-4-yl)phenyl)-carbamate (3)

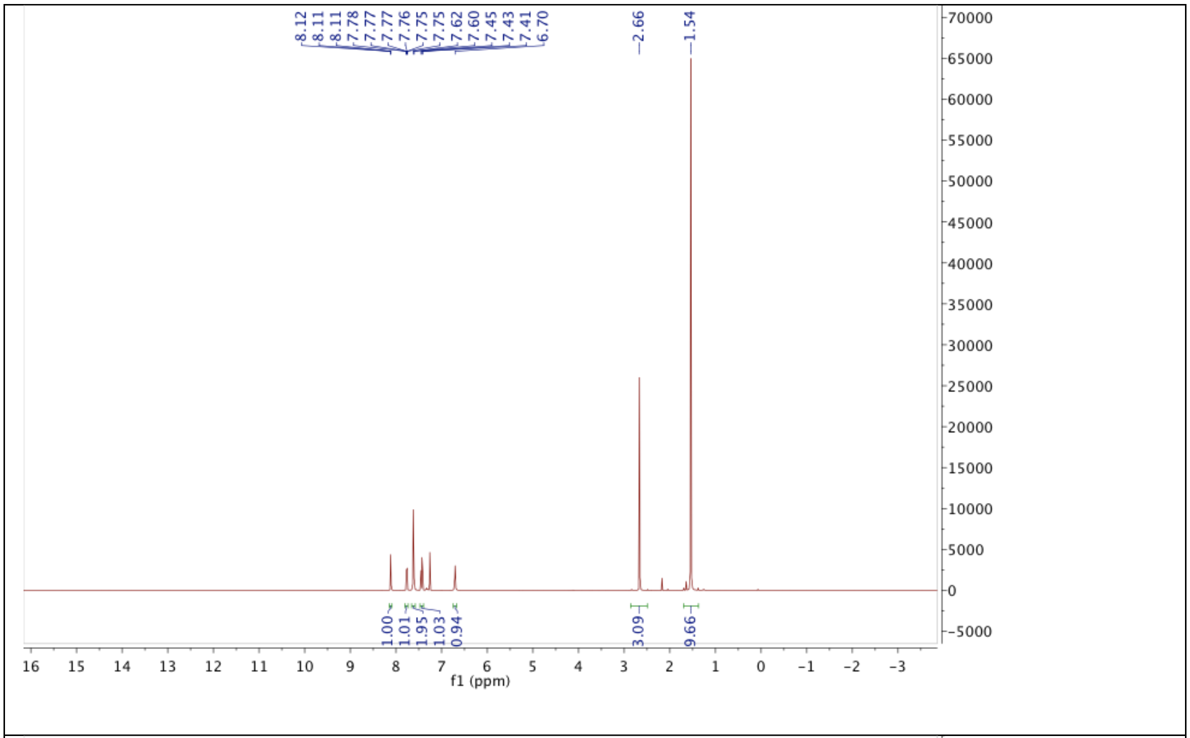

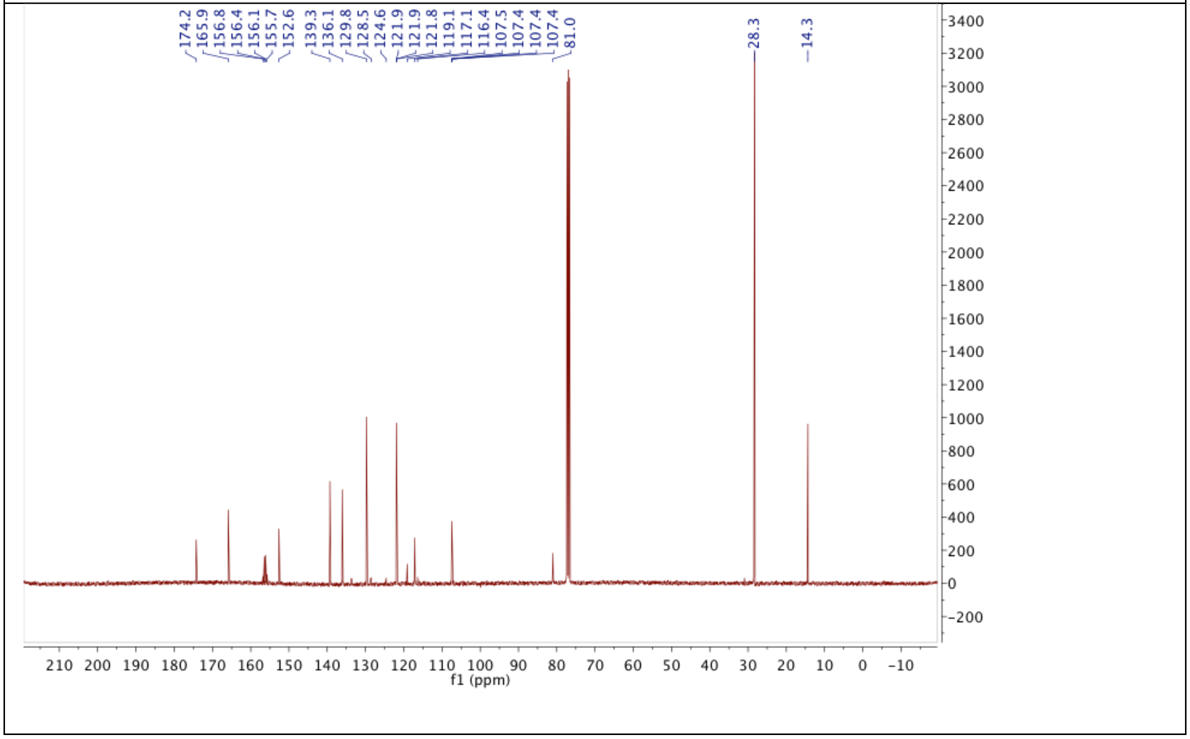

2.2. *tert*-Butyl (3-(2-(methylsulfonyl)-6-(trifluoromethyl)pyrimidin-4-yl)phenyl)-carbamate (4)

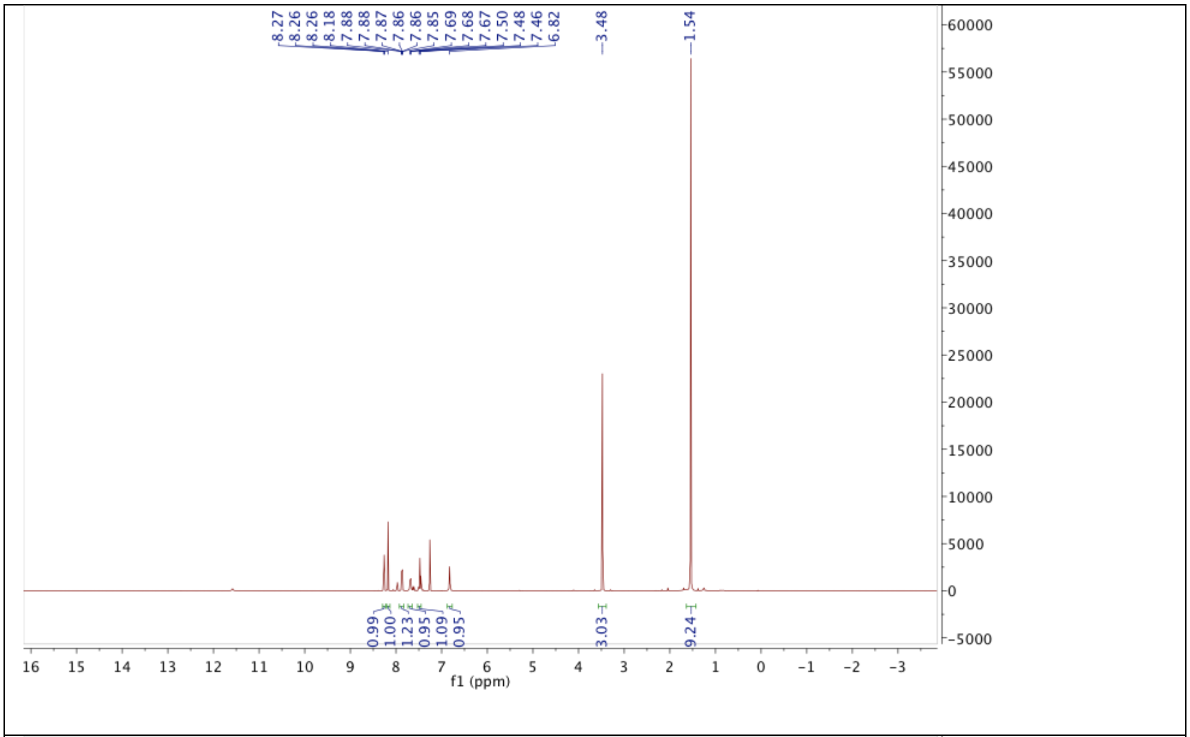

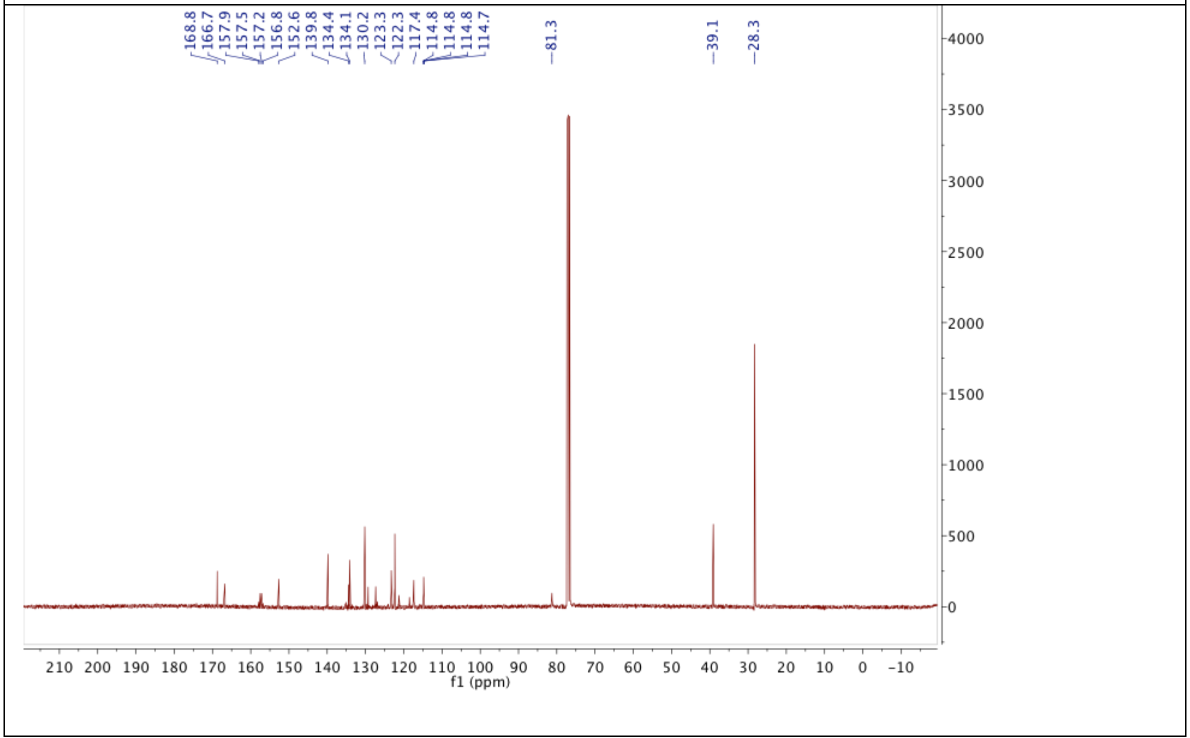

2.3. *tert*-Butyl (3-(2-(ethylthio)-6-(trifluoromethyl)pyrimidin-4-yl)phenyl)-carbamate (5)

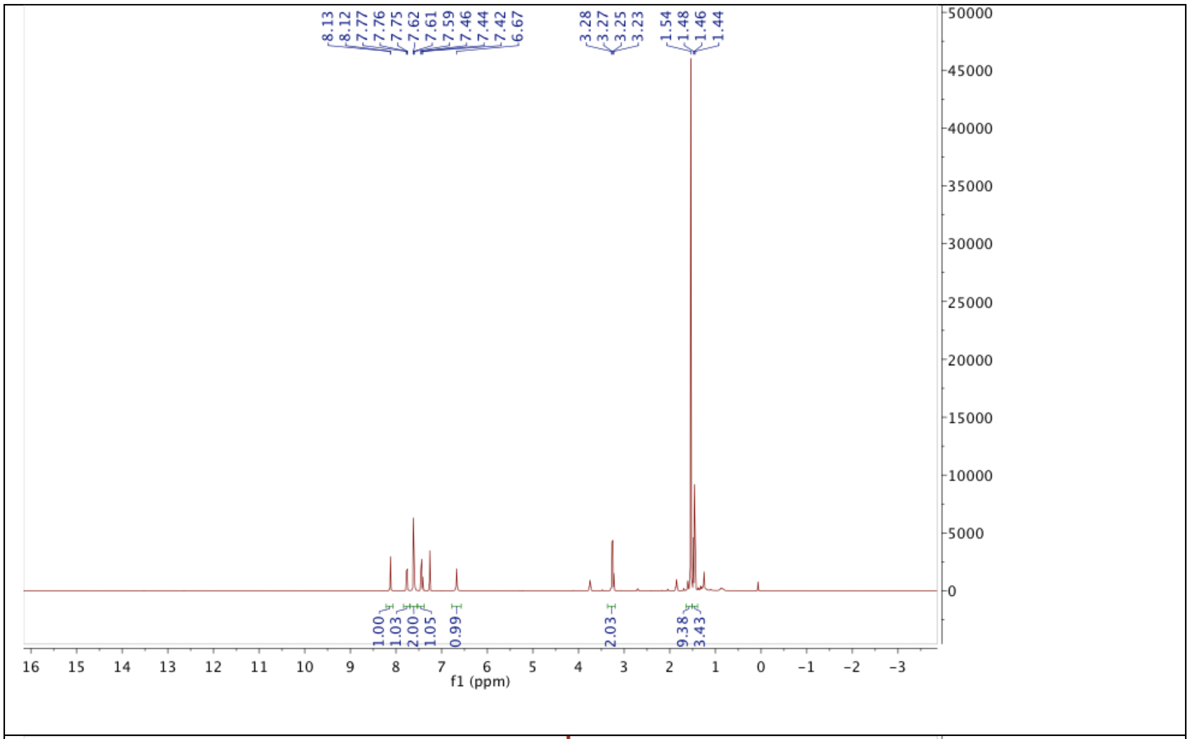

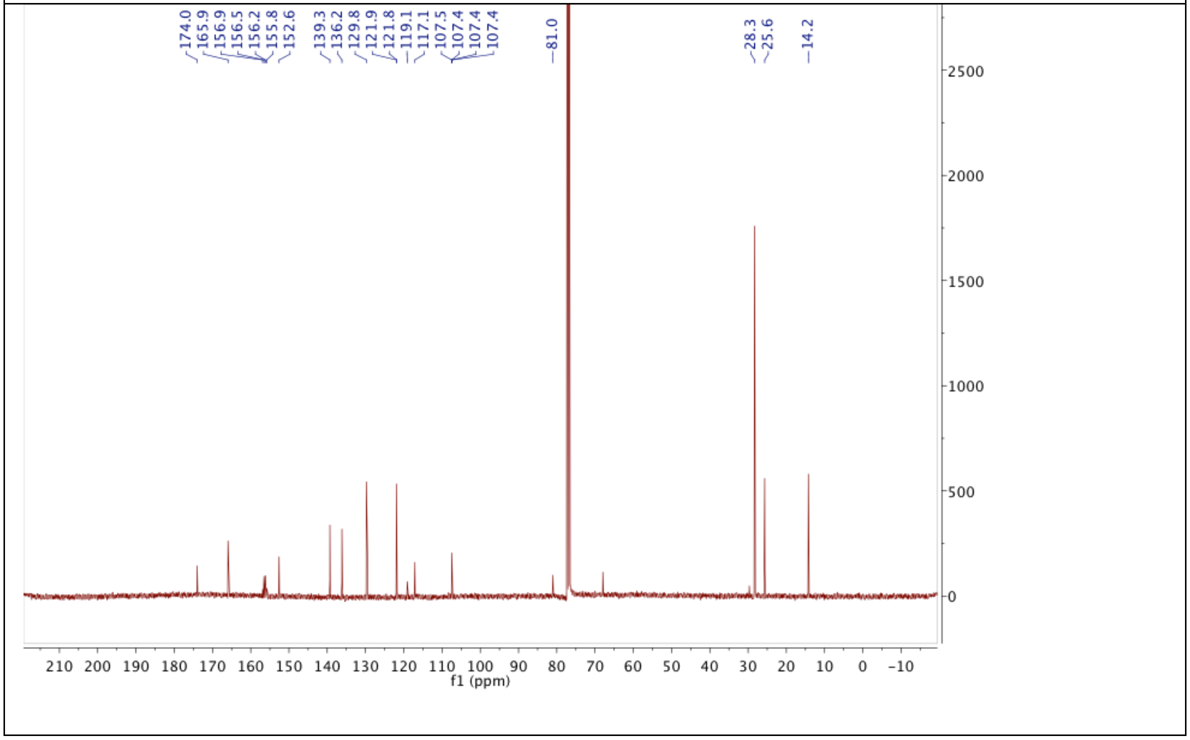

2.4. *tert*-Butyl (3-(2-(ethylsulfinyl)-6-(trifluoromethyl)pyrimidin-4-yl)phenyl)-carbamate (6)

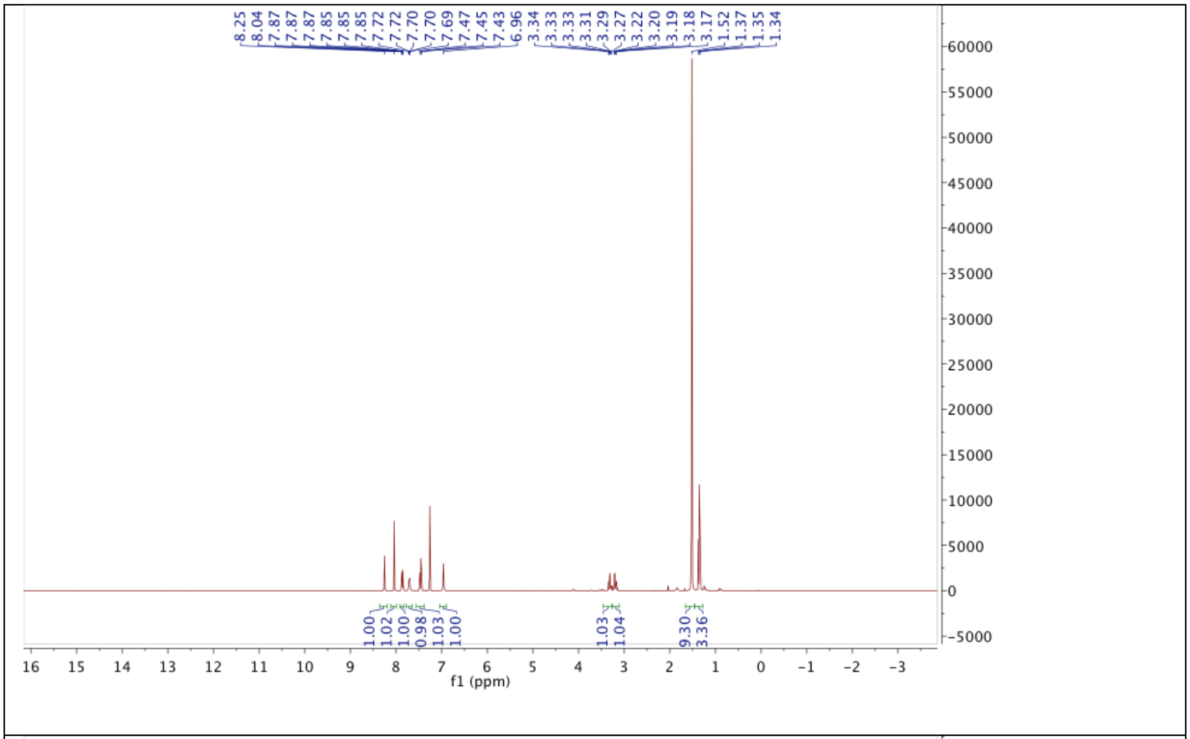

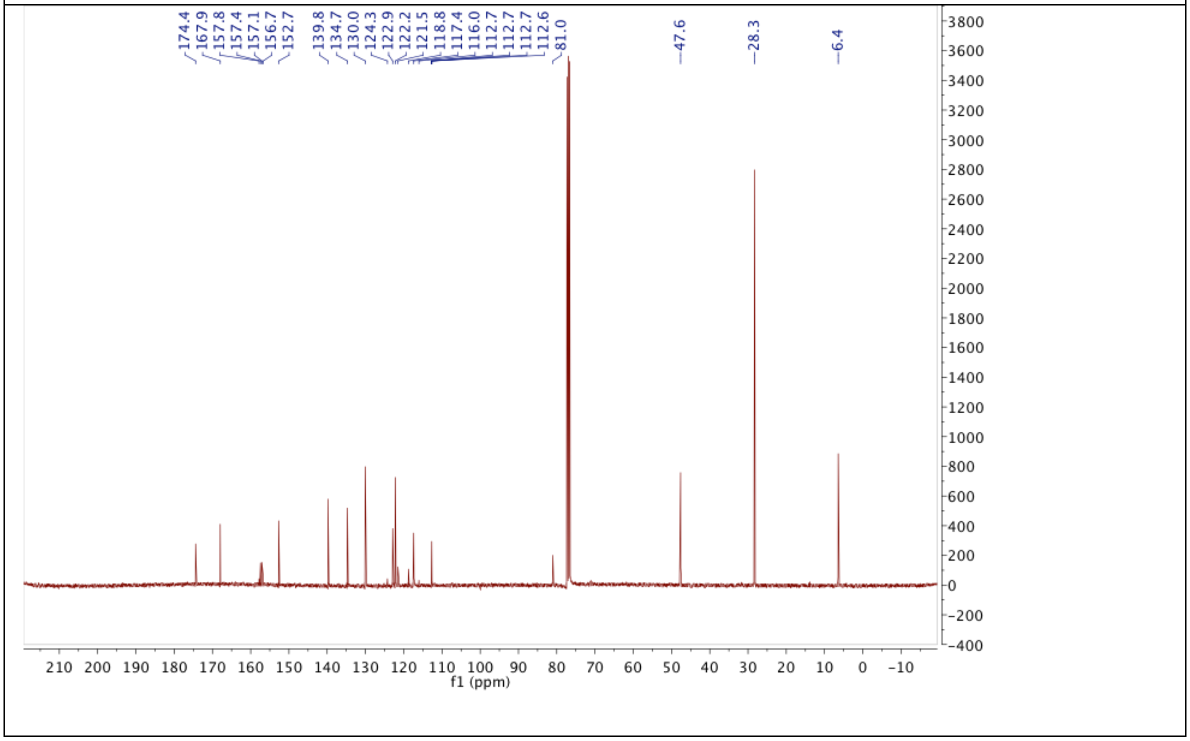

2.5. (*E*)-2-(Ethylsulfinyl)-4-(3-(phenyldiazenyl)phenyl)-6-(trifluoromethyl)-pyrimidine (PhotoETP)

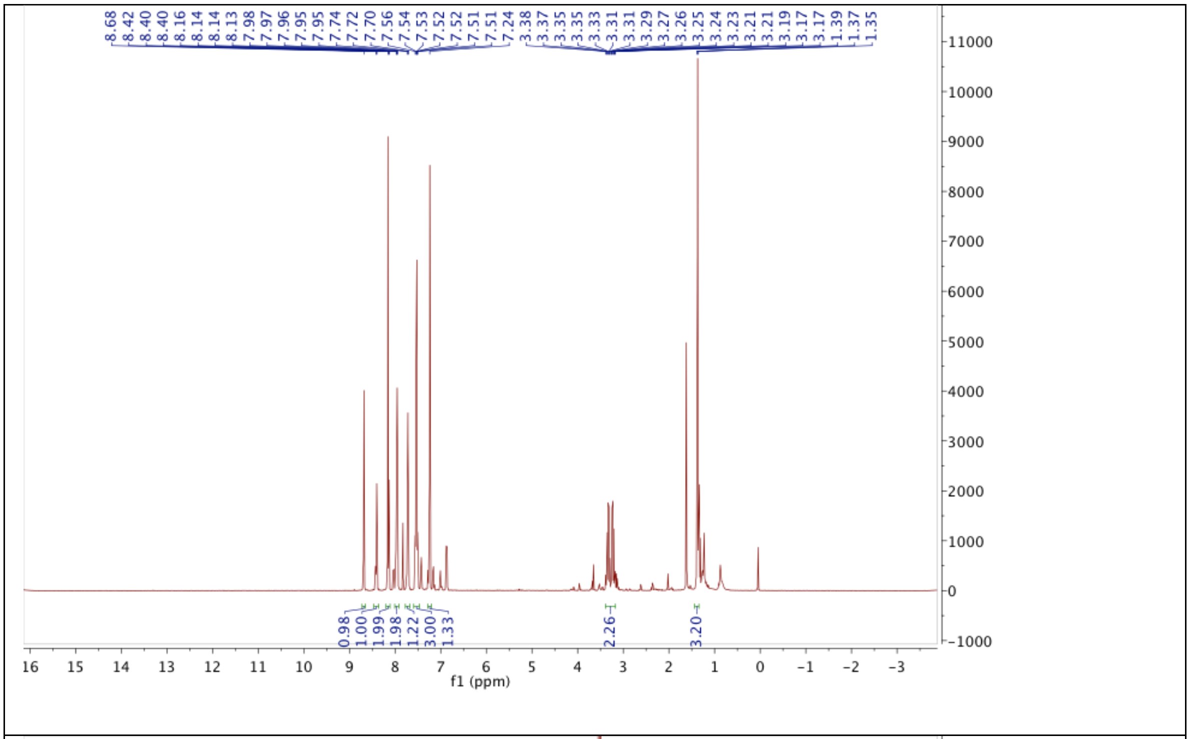

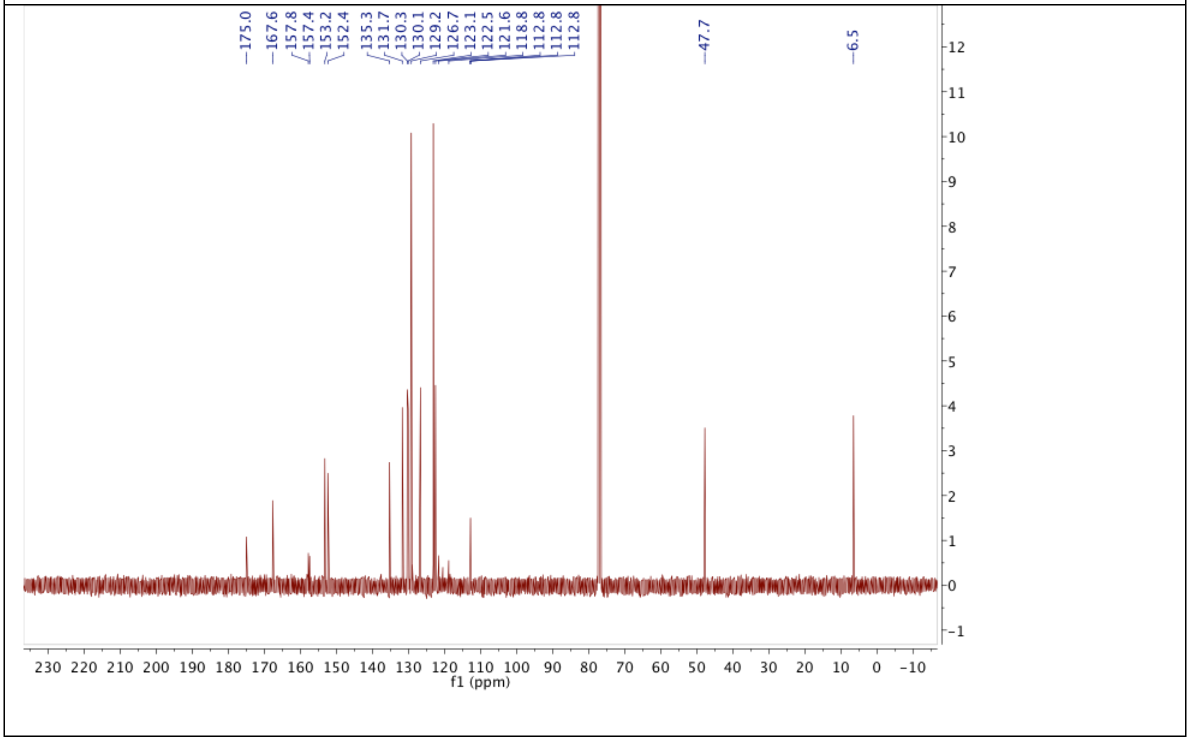

2.6. 4-(3-(Benzyloxy)phenyl)-2-((ethylthio)oxy)-6-(trifluoromethyl)pyrimidine (8)

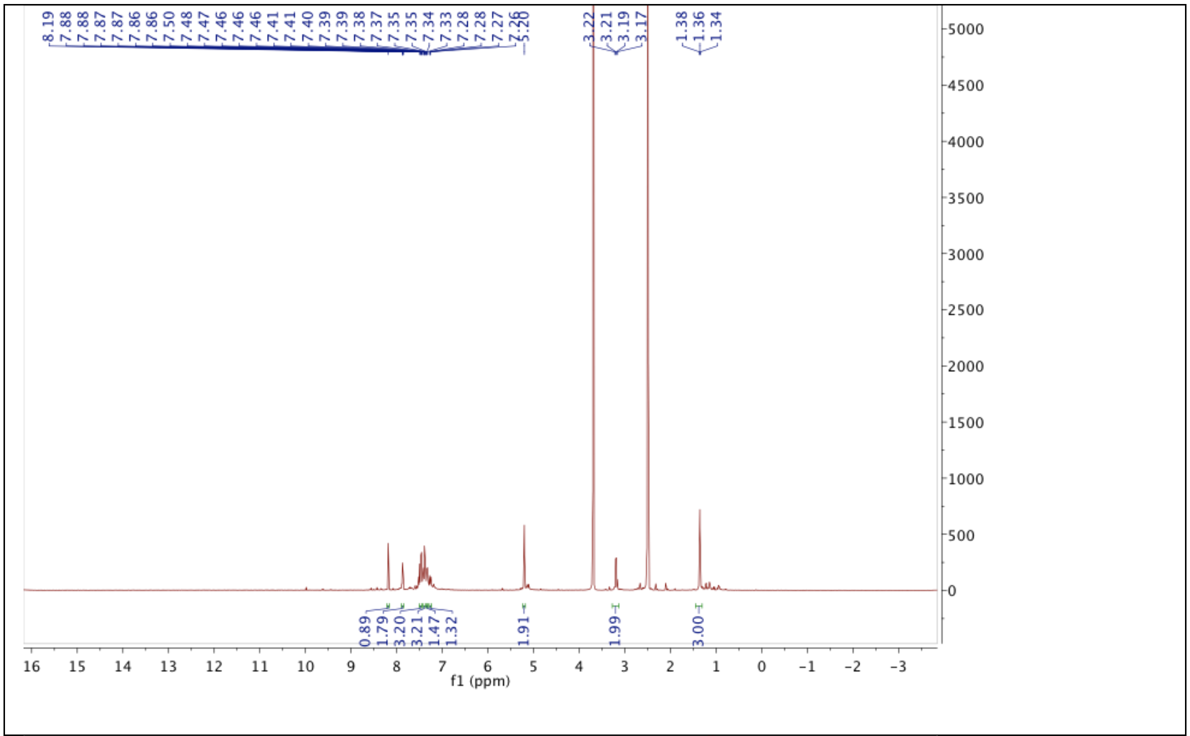

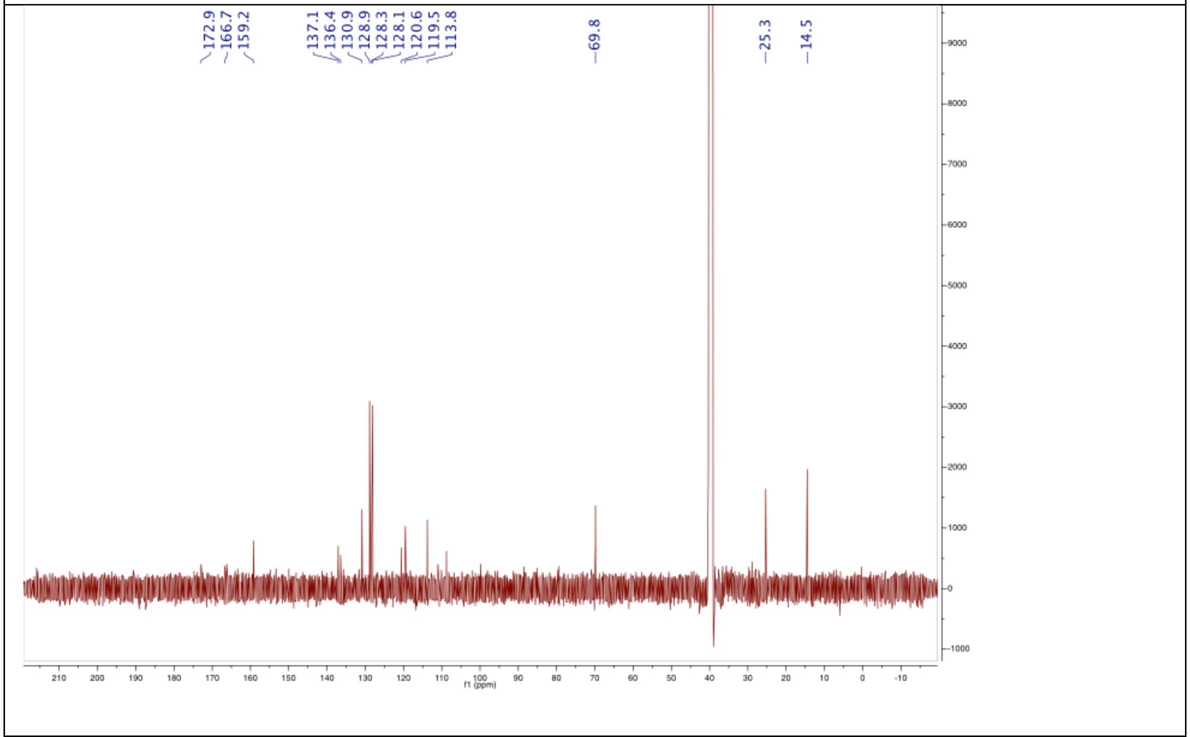

2.7. 4-(3-(Benzyloxy)phenyl)-2-(ethylsulfinyl)-6-(trifluoromethyl)pyrimidine (BETP)

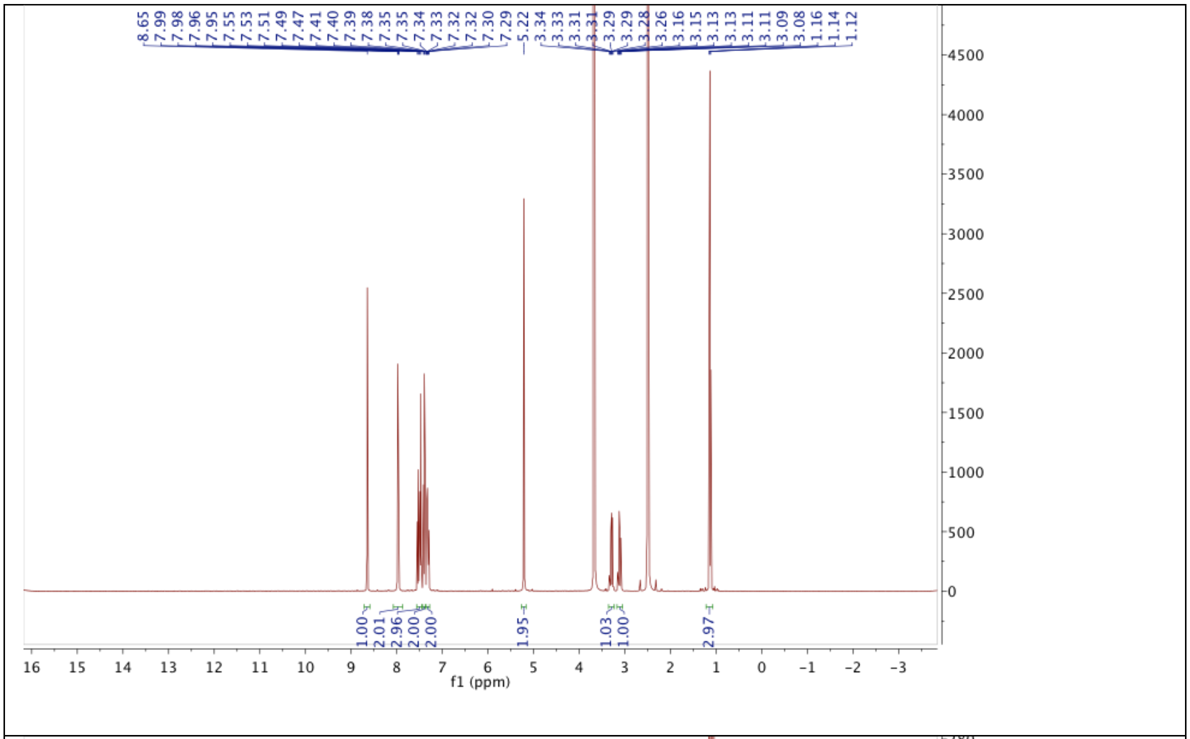

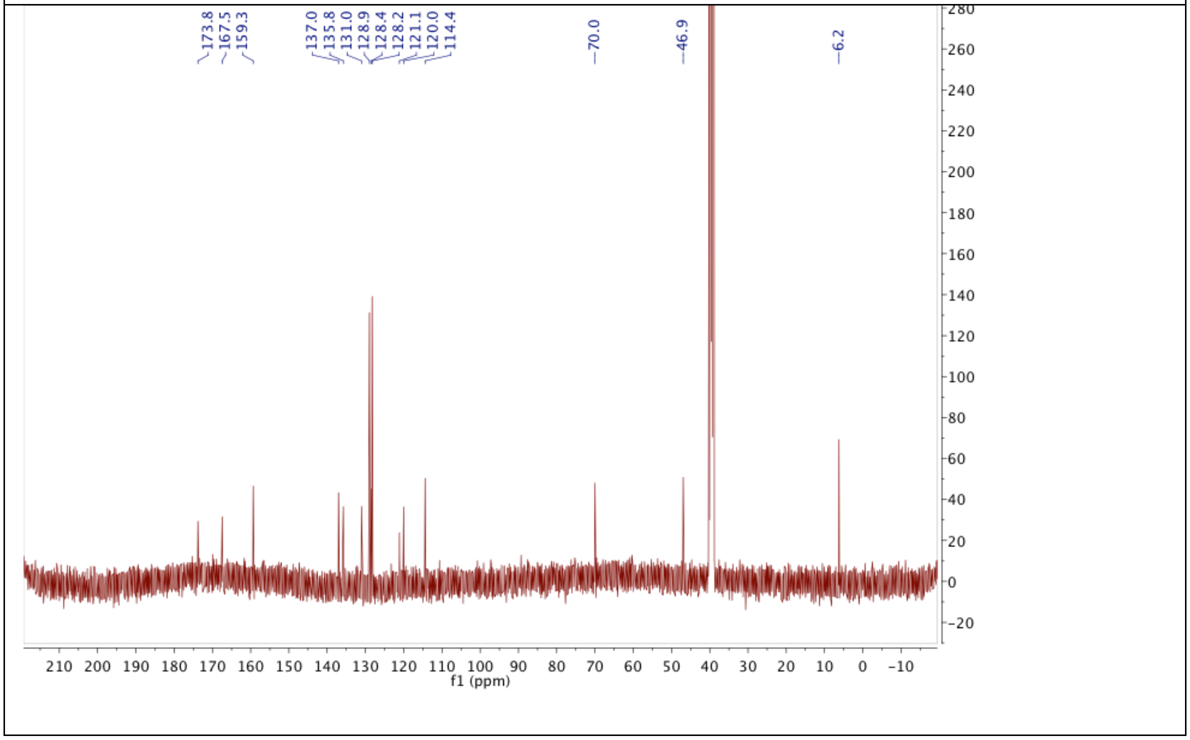

3. *X-ray crystallographic data*

3.1. *tert*-Butyl (3-(2-(methylthio)-6-(trifluoromethyl)pyrimidin-4-yl)phenyl)-carbamate (3)

Supplementary Table 1: Crystallographic data of 3

	**3**
net formula	$C_{17}H_{18}F_3N_3O_2S$
M_r/g mol^{-1}	385.405
crystal size/mm	0.143 × 0.102 × 0.057
T/K	173(2)
radiation	MoKα
diffractometer	'Oxford XCalibur'
crystal system	triclinic
space group	'P -1'
a/Å	8.7736(8)
b/Å	10.0359(9)
c/Å	10.7592(10)
α/°	78.559(8)
β/°	82.980(8)
γ/°	81.966(8)
V/Å^3	915.05(15)
Z	2
calc. density/g cm^{-3}	1.3988(2)
μ/mm^{-1}	0.222
absorption correction	'multi-scan'
transmission factor range	0.98609–1.00000
refls. measured	4669
R_{int}	0.0285
mean σ(*I*)/*I*	0.0873
θ range	4.266–25.345
observed refls.	1971
x, *y* (weighting scheme)	0.0398, 0.00
hydrogen refinement	mixed
refls in refinement	3299
parameters	243
restraints	1
$R(F_{obs})$	0.0569
$R_w(F^2)$	0.1293
S	1.012
shift/error$_{max}$	0.001
max electron density/e Å^{-3}	0.270
min electron density/e Å^{-3}	−0.268
CCDC	1420306

3.2. (*E*)-2-Methoxy-4-(3-(phenyldiazenyl)phenyl)-6-(trifluoromethyl)pyrimidine (7)

Supplementary Table 2: Crystallographic data of 7

	**7**
net formula	$C_{18}H_{13}F_3N_4O$
M_r/g mol^{-1}	358.32
crystal size/mm	0.120 × 0.020 × 0.020
T/K	100(2)
radiation	MoKα
diffractometer	'Bruker D8Venture'
crystal system	monoclinic
space group	'P 21/n'
a/Å	12.6204(6)
b/Å	7.8344(4)
c/Å	16.4847(8)
α/°	90
β/°	98.7025(15)
γ/°	90
V/Å^3	1611.13(14)
Z	4
calc. density/g cm^{-3}	1.477
μ/mm^{-1}	0.119
absorption correction	multi-scan
transmission factor range	0.9002–0.9580
refls. measured	36444
R_{int}	0.0833
mean σ(I)/I	0.0374
θ range	3.070–25.37
observed refls.	2004
x, y (weighting scheme)	0.0448, 1.0201
hydrogen refinement	constr
refls in refinement	2956
parameters	236
restraints	0
$R(F_{obs})$	0.0429
$R_w(F^2)$	0.1100
S	1.031
shift/error$_{max}$	0.001
max electron density/e Å^{-3}	0.293
min electron density/e Å^{-3}	−0.250
CCDC	1420305

4. *Experimental methods*

Isolation of rodent islets

C57BL6 mice were maintained in a specific pathogen-free facility under a 12 h light–dark cycle with ad libitum access to water and food. Animals were euthanized by cervical dislocation and collagenase solution (1 mg/mL) injected into the bile duct (clamped at the duodenal entrance), before digestion of the pancreas and isolation of islets using Histopaque gradient separation. Islets were cultured overnight in solution containing Roswell Park Memorial Institute (RPMI) medium supplemented with 10% foetal calf serum and 100 U/mL penicillin and 100 μg/mL streptomycin. All procedures were regulated by the Home Office according to the Animals (Scientific Procedures) Act 1986 of the United Kingdom (PPL 70/7349), and study approval granted by the Animal Welfare and Ethical Review Body of Imperial College. No randomization was used for animal experimentation, since mice were only used as tissue donors.

Cell lines

Chinese Hamster Ovary (CHO) cells stably expressing the GLP-1R were cultured in Dulbecco's Modified Eagle's Medium (DMEM) supplemented with 10% FBS, 2.5 mM L-glutamine, 1 x non-essential amino acids, 25 mM HEPES, 100 U/mL penicillin and 100 μg/mL streptomycin. MIN6 cells (a kind gift from Dr Jun-ichi Miyazaki, Osaka University) were cultured in DMEM supplemented with 15% FBS, 10 mM glutamine, 20 mM HEPES, 0.0005% β-mercaptoethanol, 100 U/mL penicillin and 100 μg/mL streptomycin. All cells were regularly mycoplasma tested.

Calcium imaging

Islets were loaded with Fluo2 (10 μM) for 45 min before performing functional multicellular Ca^{2+}-imaging (fMCI) using a Nipkow spinning disk system (Yokogawa CSU-10).[3] Excitation was performed using a solid state λ = 491 nm laser and emitted signals recorded from λ = 500-550 nm using a Hammamatsu ImageEM 9100-13 EM-CCD camera. Illumination to induce photoswitching was performed using either a λ = 440 nm laser or the epifluorescent port of the microscope to deliver λ = 350 ± 20 nm. In

all cases, HEPES-bicarbonate buffer was used, containing in mM: 120 NaCl, 4.8 KCl, 24 $NaHCO_3$, 0.5 Na_2HPO_4, 5 HEPES, 2.5 $CaCl_2$, 1.2 $MgCl_2$. Drugs and D-glucose were added as indicated.

High-throughput Ca^{2+} assays were performed in MIN6 cells using a DiscoverX HitHunter Calcium No Wash PLUS assay according to the manufacturer's instructions, together with Hank's Buffered Saline Solution (Invitrogen). Treatments were automatically delivered at 37 °C using a BMG NOVOstar platereader, with illumination provided λ = 340 ± 10 nm or λ = 450 ± 10 nm using an inbuilt monochromator. Absorbance and emission were read at λ = 488 nm and λ = 525 nm, respectively.

cAMP assays

Cellular cAMP levels were determined using a Cisbio dynamic 2 kit according to the manufacturer's instructions and expressed as a relative change (% max-min). Treatments were applied for 15 min in the presence of 100 µM IBMX with and without illumination at λ = 350 ± 30 nm using a UVP transilluminator, before cell lysis to extract cytoplasmic cAMP.

Insulin assays

Six islets were incubated for 30 min in Krebs-HEPES-bicarbonate solution containing in mM: 130 NaCl, 3.6 KCl, 1.5 $CaCl_2$, 0.5 $MgSO_4$, 0.5 NaH_2PO_4, 2 $NaHCO_3$, 10 HEPES and 0.1% (wt/vol) bovine serum albumin, pH 7.4. Treatments were applied as indicated and photoswitching performed using a BMG Fluostar Optima platereader, as for Ca^{2+} assays. Insulin concentrations in the supernatant were measured using a Cisbio HTRF assay according to the low-range (sensitive) protocol.

Necrosis and apoptosis assays

Necrosis was determined in control, dimethyl sulfoxide (DMSO), BETP or PhotoETP-treated islets following incubation with calcein-AM (3 µM; live) and propidium iodide (2.5 µM; dead). Absorbance/emission was detected at λ = 491/525 nm (calcein) and λ = 561/620 nm (propidium iodide), and the area of dead:live cells calculated as a ratio.

Apoptosis was assessed using either a DeadEnd Fluorimetric TUNEL System staining kit (Promega). Proteolytic cleavage was determined using a rabbit polyclonal antibody against cleaved caspase 3 (Cell Signaling Technology # 9661; 1:400). The apoptotic/proteolytic cell mass was calculated as a fraction area versus the total cell mass.

Statistics

Data normality was assessed using the D'Agostino-Pearson test. Non-multifactorial comparisons were made using Student's t-test. Multifactorial comparisons were made using one-way ANOVA followed by Bonferonni's post-hoc test according to degrees of freedom. Log-transformed concentration-response curves were fitted using non-linear regression and a 3- or 4- parameter fit. All analyses were conducted using Graphpad Prism (Graphpad Software) and IgorPro. Results were considered significant at $P<0.05$.

5. *BETP photodegradation*

During control experiments in which BETP itself was exposed to UV light, we noticed appearance of a second prominent peak in the LC-MS profile (Figure S5A,B). After isolation and chemical characterization, the photodegraded compound was found to be the rearranged sulfenic ester **8** (Figure S5A,B). Investigation of the reaction by ^{1}H-NMR (Figure S5C) revealed a clean process, wherein i) the triplet corresponding to the terminal methyl group shifts downfield, ii) the multiplets of the diastereotopic methylene protons merge to a quartet, and iii) >78% yield with no characterizable side products was obtained (Figure S6). Zero-order kinetics for the formation of **8** were observed (k_{obs} = 0.461 ± 0.036 µmol/h) (Figure S5D), consistent with an intramolecular rearrangement. While the innate electrophilicity of BETP towards nucleophiles has been studied and Meisenheimer complexes discussed,[4] rearrangements of this type have not been described to the best to our knowledge.

As expected, this photodegradation process rendered BETP less effective as an allosteric modulator. Whereas BETP left-shifted the cAMP dose response to GLP-1(9-36)NH_2 in CHO-GLP-1R cells (EC_{50} (BETP) = 60.8 nM), exposure to UV during the same assay dramatically decreased the half maximal (EC_{50} (BETP + UV) = 393.4 nM) and maximal response values (35.9 ± 1.6 % of the max) (Figure S7A). This was not due to photodegradation of GLP-1(9-36)NH_2 itself, since BETP responses were largely unaffected by prior UV-illumination of the incretin metabolite (Figure S7B). Similarly, cells exposed to UV retained full responsiveness to the GLP-1R agonist exendin 4, suggesting normal signaling/binding capacity (Fig. S7C). Lastly, BETP did not undergo photodegradation in response to white light (*i.e.* benchtop conditions) (Fig. S8).

6. *Supporting Figures*

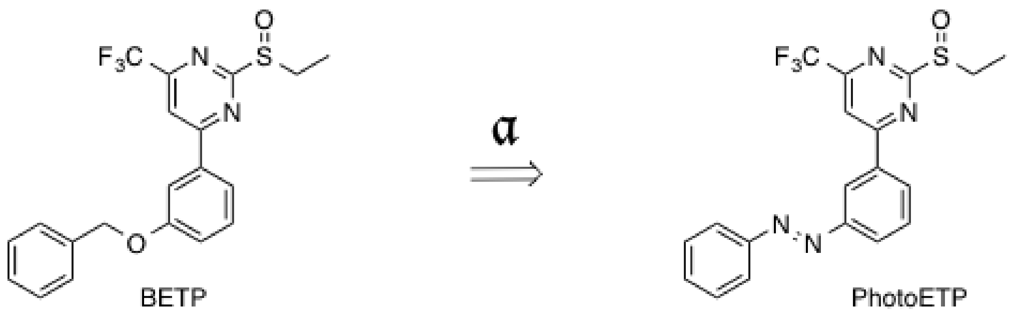

Supplementary Figure 1. Azologization (a) of BETP to **PhotoETP** displaces the benzyl ether with a diazenyl phenyl group.

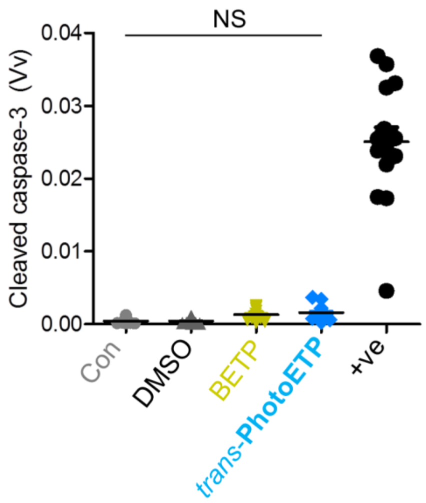

Supplementary Figure 2. Cleaved caspase-3 protein expression is unaffected following 3 hr incubation of MIN6 cells with BETP or **PhotoETP** (both 50 µM) (+ve; staurosporine) (*n* = 3). Bar represents the mean.

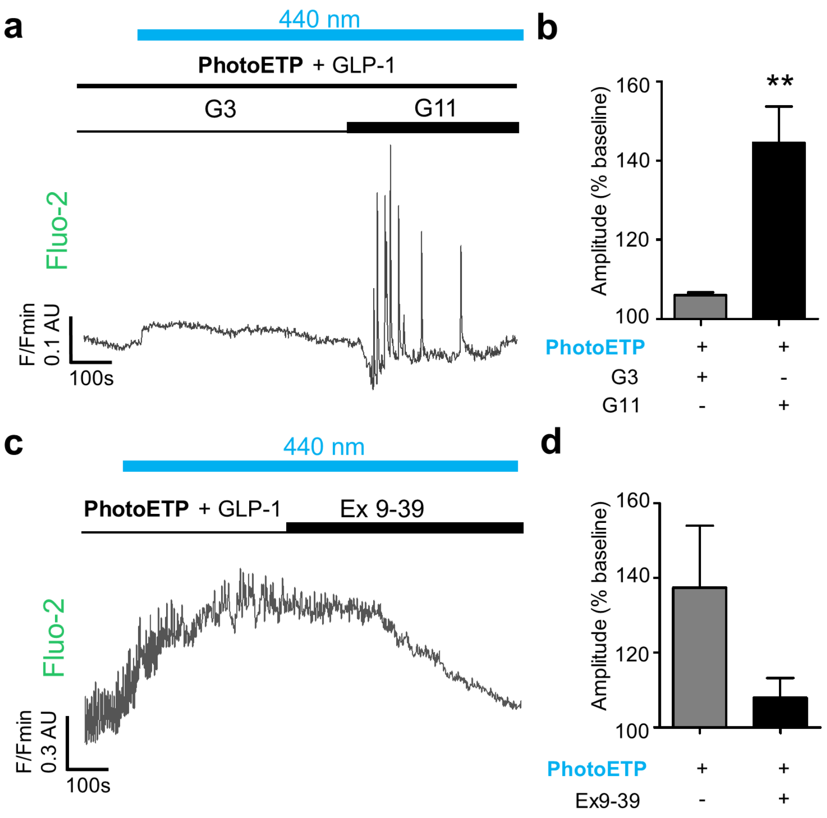

Supplementary Figure 3. a,b) **PhotoETP** responses are glucose-dependent in islets (G3, 3 mM glucose; G11, 11 mM glucose) (n = 7-9 recordings). c,d) Application of the specific GLP-1R antagonist exendin 9-39 100 nM (Ex 9-39) reverses the effects of **PhotoETP** on intracellular free Ca^{2+} rises in islets (n = 3 recordings). In all cases, GLP-1(7-36)NH_2 was co-applied at 10 nM in the presence of 8-11 mM glucose unless otherwise indicated. **PhotoETP** was applied at 50 µM. **P<0.01, Student's t-test. Values represent mean ± s.e.m.

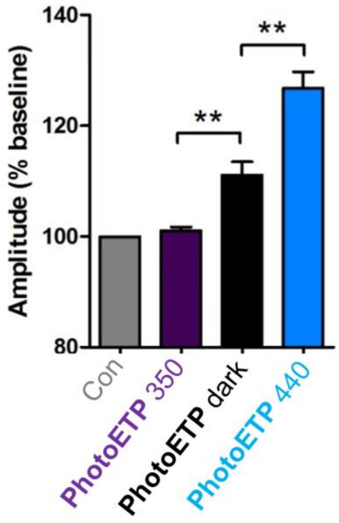

Supplementary Figure 4. PhotoETP allows graded Ca^{2+} responses in response to illumination, with UV being less effective than dark and 440 nm (*n* = 5 recordings). In all cases, GLP-1(7-36)NH_2 co-applied at 10 nM in the presence of 11 mM glucose unless otherwise indicated. **PhotoETP** was applied at 50 µM. **P<0.01, one-way ANOVA. Values represent mean ± s.e.m.

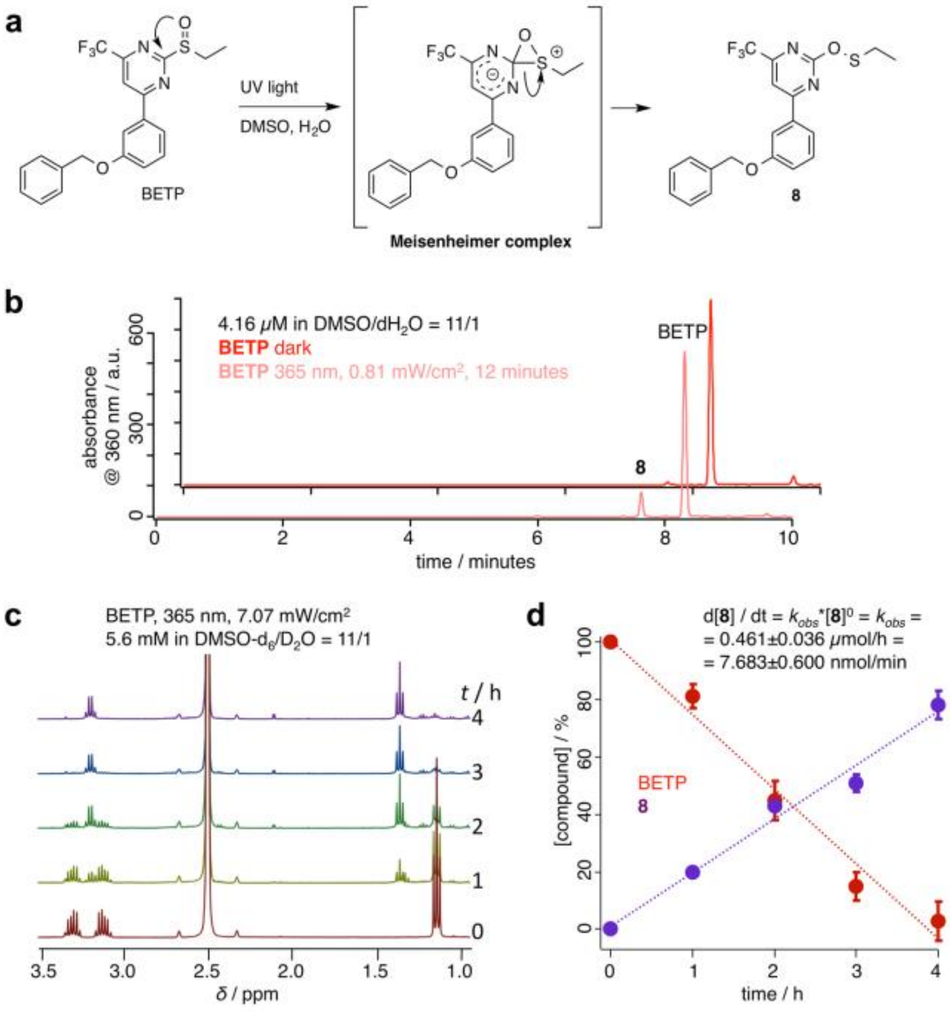

Supplementary Figure 5. Photodegradation of BETP using UV light. a) Proposed degradation mechanism. b) LC-MS trace that shows appearance of product **8**. c) ^{1}H-NMR shows complete disappearance of BETP over 4 h. d) Zero-order kinetics suggest an intramolecular degradation mechanism.

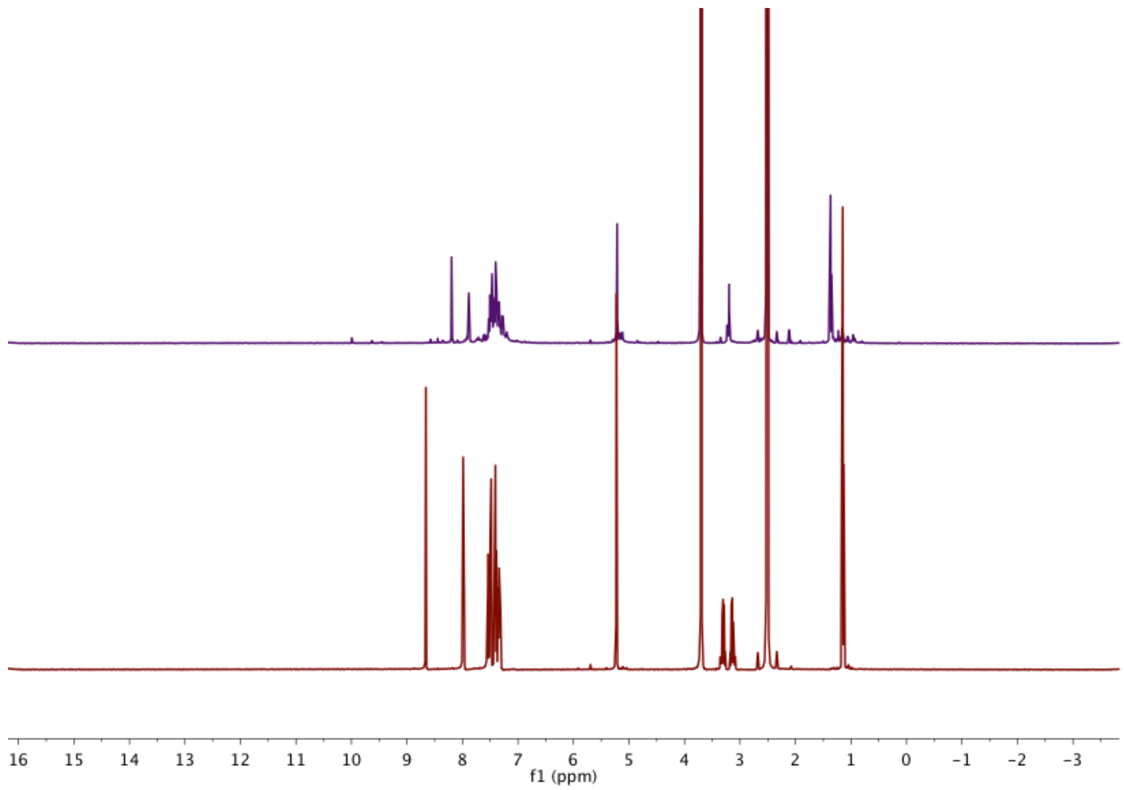

Supplementary Figure 6. Photodegradation of BETP to **8** by NMR-spectroscopic kinetics. Full ^{1}H-NMR spectral overlay of BETP before irradiation with 365 nm (red, bottom) and after 4 hours (purple, top) in DMSO-d_6/D_2O (11/1).

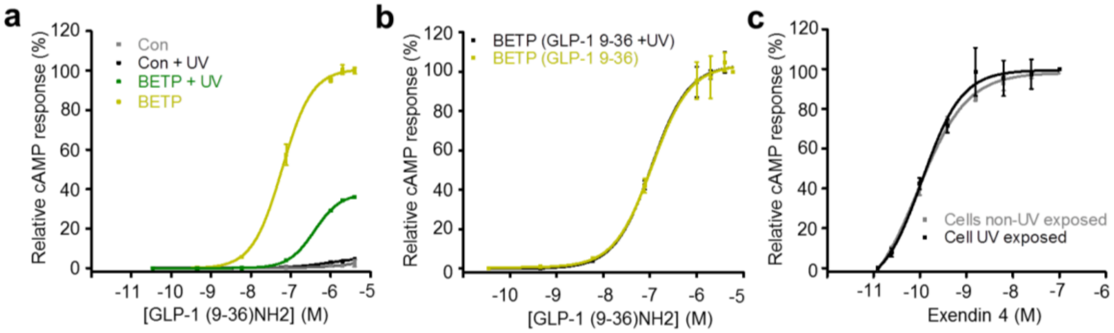

Supplementary Figure 7. Photodegraded BETP displays reduced activity. a) BETP exposed to λ = 350 nm (UV) is less effective (*n* = 4 repeats). b) Pre-illumination of GLP-1(9-36)NH_2 with UV does not affect responses to BETP (*n* = 3 repeats). c) Pre-illumination of CHO-GLP-1R cells with λ = 350 nm does not affect responses to exendin 4 (*n* = 3 repeats). **PhotoETP** and BETP were applied at 50 µM. Values represent mean ± s.e.m.

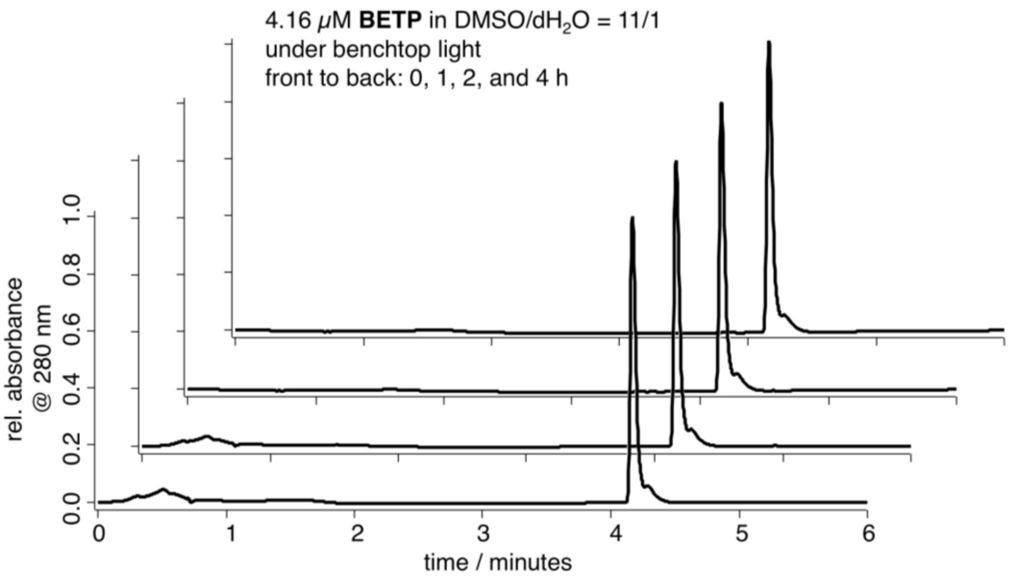

Supplementary Figure 8. BETP is not photodegraded by white light. LC-MS traces show no change in the chromatogram of BETP when a vial containing a solution of BETP was standing under ambient laboratory "benchtop" light over 0, 1, 2 and 4 hours (front to back).

7. *References*

[1] A. Altomare, M. C. Burla, M. Camalli, G. L. Cascarano, C. Giacovazzo, A. Guagliardi, A. G. G. Moliterni, G. Polidori, R. Spagna, *J. Appl. Crystallogr.* **1999**, *32*, 115-119.

[2] G. Sheldrick, *Acta Crystallographica Section A* **2008**, *64*, 112-122.

[3] aD. J. Hodson, R. K. Mitchell, E. A. Bellomo, G. Sun, L. Vinet, P. Meda, D. Li, W. H. Li, M. Bugliani, P. Marchetti, D. Bosco, L. Piemonti, P. Johnson, S. J. Hughes, G. A. Rutter, *J. Clin. Invest.* **2013**, *123*, 4182-4194; bD. J. Hodson, F. Molino, P. Fontanaud, X. Bonnefont, P. Mollard, *J. Neuroendocrinol.* **2010**, 1217-1225.

[4] H. Eng, R. Sharma, T. S. McDonald, D. J. Edmonds, J. P. Fortin, X. Li, B. D. Stevens, D. A. Griffith, C. Limberakis, W. M. Nolte, D. A. Price, M. Jackson, A. S. Kalgutkar, *Drug Metab. Dispos.* **2013**, *41*, 1470-1479.
